# Emerging Trends in Fast MRI Using Deep-Learning Reconstruction on Undersampled k-Space Data: A Systematic Review

**DOI:** 10.3390/bioengineering10091012

**Published:** 2023-08-26

**Authors:** Dilbag Singh, Anmol Monga, Hector L. de Moura, Xiaoxia Zhang, Marcelo V. W. Zibetti, Ravinder R. Regatte

**Affiliations:** Center of Biomedical Imaging, Department of Radiology, New York University Grossman School of Medicine, New York, NY 10016, USA; anmol.monga@nyulangone.org (A.M.); hector.lisedemoura@nyulangone.org (H.L.d.M.); xiaoxia.zhang@nyulangone.org (X.Z.); marcelo.wustzibetti@nyulangone.org (M.V.W.Z.)

**Keywords:** Magnetic Resonance Imaging, parallel MRI, compressive sensing, k-space, deep learning, deep MRI reconstruction, Deep Bayesian Learning, deep dictionary learning, acquisition time reduction, fast MRI

## Abstract

Magnetic Resonance Imaging (MRI) is an essential medical imaging modality that provides excellent soft-tissue contrast and high-resolution images of the human body, allowing us to understand detailed information on morphology, structural integrity, and physiologic processes. However, MRI exams usually require lengthy acquisition times. Methods such as parallel MRI and Compressive Sensing (CS) have significantly reduced the MRI acquisition time by acquiring less data through undersampling k-space. The state-of-the-art of fast MRI has recently been redefined by integrating Deep Learning (DL) models with these undersampled approaches. This Systematic Literature Review (SLR) comprehensively analyzes deep MRI reconstruction models, emphasizing the key elements of recently proposed methods and highlighting their strengths and weaknesses. This SLR involves searching and selecting relevant studies from various databases, including Web of Science and Scopus, followed by a rigorous screening and data extraction process using the Preferred Reporting Items for Systematic Reviews and Meta-Analyses (PRISMA) guidelines. It focuses on various techniques, such as residual learning, image representation using encoders and decoders, data-consistency layers, unrolled networks, learned activations, attention modules, plug-and-play priors, diffusion models, and Bayesian methods. This SLR also discusses the use of loss functions and training with adversarial networks to enhance deep MRI reconstruction methods. Moreover, we explore various MRI reconstruction applications, including non-Cartesian reconstruction, super-resolution, dynamic MRI, joint learning of reconstruction with coil sensitivity and sampling, quantitative mapping, and MR fingerprinting. This paper also addresses research questions, provides insights for future directions, and emphasizes robust generalization and artifact handling. Therefore, this SLR serves as a valuable resource for advancing fast MRI, guiding research and development efforts of MRI reconstruction for better image quality and faster data acquisition.

## 1. Introduction

Magnetic Resonance Imaging (MRI) is a method of obtaining detailed images of the internal structure of the body by using magnetic fields and radio waves. Several medical conditions can be diagnosed, treated, and detected using these images [1]. However, a well-known issue is its “long scan time”, which requires patients to remain still for a long time, sometimes almost an hour. [2]. This process would be difficult for patients, particularly for those with claustrophobia, pain, and difficulties to stay still, ultimately leading to scan failure. Long scan time also reduces scanner usage, reducing broad access to this exam and increasing costs. Therefore, minimizing the scan time is highly significant in clinical studies and very important in clinical practice. This can be achieved using undersampled k-space data which accelerates the MRI scanning process [3,4].

**Figure 1:**
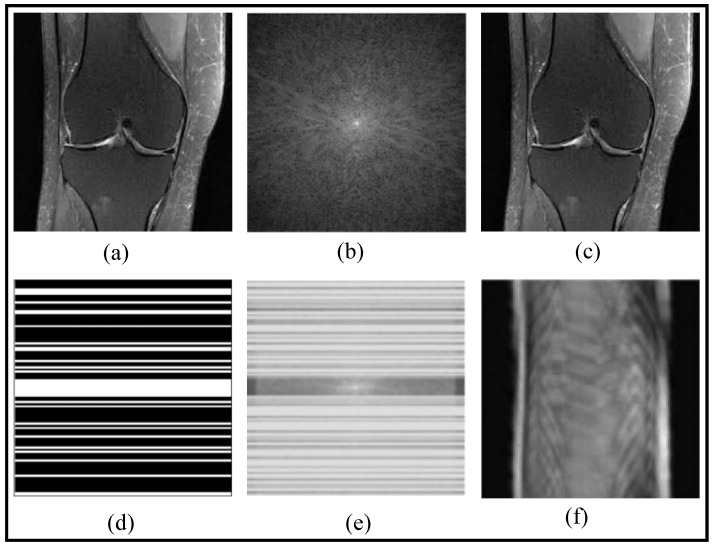
Fast MRI Knee Image Reconstruction: From Fully Sampled to Aliased Images: (**a**) Reconstructed MRI obtained from DL method (*X*), (**b**) $y^{\mathrm{Full}}$, (**c**) $X^{\mathrm{Full}}$, (**d**) *M*, (**e**) *y*, and (**f**) $X^{\mathrm{aliased}}$ (adapted with changes from [5]).

In Figure 1, a fast MRI reconstruction process is demonstrated through a set of notations. The knee image is represented as *X*, and its corresponding fully sampled k-space data is denoted as $y^{\mathrm{Full}}$, which allows obtaining the fully reconstructed image $X^{\mathrm{Full}}$. To accelerate MRI acquisition, a sampling mask, denoted as *M*, is applied to selectively acquire k-space data, resulting in the undersampled k-space data *y*. Using this undersampled data and the sampling mask, an aliased reconstructed image $X^{\mathrm{aliased}}$ is obtained, which is usually of no practical clinical use. However, fast reconstruction methods can recover a better image *X*, removing the artifacts of the undersampling acquisition.

Many MRI reconstruction techniques such as Sensitivity Encoding (SENSE) [6], Generalized Autocalibrating Partially Parallel Acquisitions (GRAPPA) [7], and Self-Consistent Parallel Imaging Reconstruction (SPIRiT) [8] utilize undersampled k-space data and information obtained from multiple radio-frequency (RF) receiver coils to reconstruct high-quality MR images. These techniques use more data from multiple coils with different spatial sensitivities, filling the missing k-space data by exploiting the correlation between k-space data from different RF receiver coils. They enable improved spatial resolution, minimize motion artifacts, and enable faster clinical workflows by leveraging parallel imaging principles and iterative reconstruction models [6,7,9]. Joint Compressive Sensing and Parallel imaging (CS-P) methods combine the power of both techniques to enhance the quality and efficiency of MRI reconstruction. By jointly exploiting the sparse representation of MRIs and the parallel acquisition of data, these methods enable high-quality reconstruction from undersampled measurements [10,11,12,13].

Reconstruction accuracy can also be affected by noise in the acquired data [14,15,16]. Parallel MRI increases localized noise, usually defined by the g-factor of the specific method [17,18,19,20]. CS approaches improve it by using regularization filters that smooth the images, however, they are computationally costly and they rely on the proper selection of the regularization penalty and their parameters.

In recent years, Deep Learning (DL) has emerged as a powerful tool for improving the quality and speed of MRI reconstruction [1,2,5,21,22,23,24,25,26,27,28,29,30,31]. By learning complex mappings using undersampled k-space data and fully-sampled images, DL can reconstruct high-quality images from limited data. It has been demonstrated that DL networks such as Convolutional Neural Networks (CNNs) [32], Variational Networks (VN) [33,34], and Generative Adversarial Network (GAN) [35,36,37] significantly reduce undersampling artifacts, improve MRI quality, and enabling fast MR imaging.

MRI researchers have leveraged DL to efficiently estimate quantitative tissue parameters from complex-valued data, including MR quantitative mapping [38], quantitative susceptibility mapping [39], and MR fingerprinting [40]. These networks incorporate the physical model of the quantitative parameters, enabling accurate mappings [41,42]. Thus, DL-based approaches in MRI reconstruction have the potential for significant benefits. However, uncertainties arising from missing k-space data points and network weights have hindered their adoption in clinical practice. To overcome the challenges associated with uncertainties in DL-based MRI reconstruction, Bayesian methods have been evolved to use deep learning [43,44,45]. These methods aim to address the uncertainties associated with undersampled reconstruction by providing spatially uncertainty maps.

The main objective of this paper is to conduct a Systematic Literature Review (SLR) on deep MRI reconstruction methods, specifically evaluating network architectures, attention mechanisms, residual learning, and loss functions. The goal is to provide valuable insights for future directions in fast MRI, with a particular emphasis on robust generalization and artifact handling. The subsequent section covers several important aspects, including the motivation behind conducting SLR, an overview of the related work in the field, data sources utilized for SLR, research questions which will be addressed throughout this SLR.

### 1.1. Motivation

DL networks have proven successful in reconstructing MRIs from limited measurements by leveraging the ability to learn effective models from sample data. This breakthrough sparked considerable interest in deep MRI reconstruction, leading to ongoing advancements in network architectures, data augmentation techniques, regularization approaches, and loss functions. Researchers continuously explore ways to enhance performance and generalizability in deep MRI reconstruction. However, deep reconstruction networks are still an evolving field of research.

Despite significant advances in deep MRI reconstruction, challenges related to accuracy and speed remain. Additionally, there is a need for further research to gain a better understanding of the underlying mechanisms involved in this technique. To address these issues, this paper presents an SLR covering the period from January 2018 to June 2023. The primary objective is to provide a comprehensive overview of recent advancements in deep MRI reconstruction. Moreover, this paper explores the challenges and opportunities in the field and offers insights into its future development. The SLR aims to enhance the current understanding of deep MRI reconstruction and serve as a guide for future research in this area.

### 1.2. Review of Related Works

In recent years, the field of deep MRI reconstruction has garnered significant attention, leading to the publication of numerous review and survey papers. Table 1 presents a comparative analysis of these papers, highlighting their categories and features, such as Compressed Sensing (CS), Super Resolution (SR), Quantitative Mapping (QM), and Magnetic Resonance Fingerprinting (MRF). It is observed that researchers primarily focus on DL and CS-based MRI reconstruction models, often overlooking important features like SR, QM, and MRF. Only two researchers have conducted SLR [2,27]. Moreover, recent advancements in Non-Cartesian reconstruction Plug-and-Play priors, Diffusion models, and Bayesian methods have been largely neglected. Therefore, this paper aims to address these gaps by considering published papers between Jan. 2018 and June 2023, covering all the discussed factors comprehensively.

### 1.3. Data Source

The article selection process for the Web of Science and Scopus databases involved specific keyword combinations related to deep learning-based MRI reconstruction. Keywords included compressive sensing, super-resolution, end-to-end DL for parametric mapping, DL-based MRF quantitative mapping, deep Bayesian, and deep dictionary learning for MRI reconstruction. The search aimed to retrieve articles focusing on these specific aspects.

**Figure 2:**
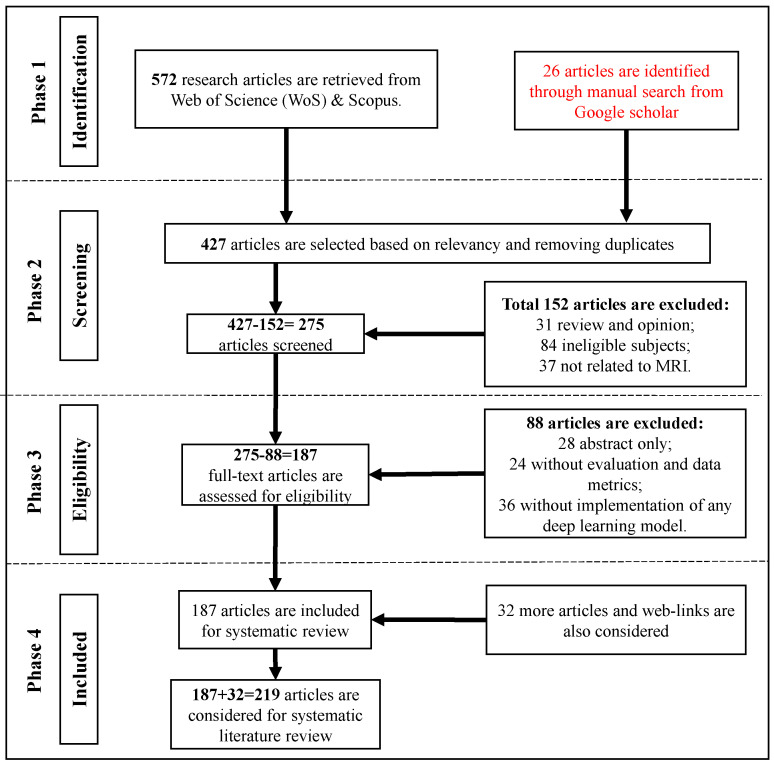
Methodology and Criteria for Inclusion and Exclusion of Research Articles from WoS and Scopus Databases: A PRISMA Guideline-Based Approach, Augmented with a Manual Search on Google Scholar.

Figure 2 illustrates a flowchart depicting the article selection process based on the guidelines of Preferred Reporting Items for Systematic Reviews and Meta-Analyses (PRISMA). It is found that in a total of 572 research articles are retrieved from Web of Science (WoS) and Scopus published between Jan. 2018 to June 2023. An additional 26 articles are identified through a manual search on Google Scholar. After removing duplicates and assessing relevancy, 427 articles are selected for further evaluation.

From these 427 articles, 275 articles are screened by excluding 152 articles due to being review and opinion papers (31), irrelevant subjects (84), or not related to MRI (37). From the remaining 275 articles to be screened for eligibility, 88 articles are removed by considering abstract-only (28), lacking evaluation and data metrics (24), and not implementing any DL model (36). In total, 187 articles from WoS and Scopus are included for SLR. Additionally, 32 more articles and web-links are consider‘ed. These articles are freely selected, some of which were published even before 2018.

### 1.4. Research Questions

This paper aims to explore the current landscape of fast MRI reconstruction using DL models on undersampled k-space data. This SLR addresses the following research questions:

(a) How do advanced network architectures, including residual learning, image representation using encoders and decoders, data-consistency layers, unrolled networks, learned activations, attention modules, plug-and-play priors, diffusion models, and Bayesian methods contribute to the development of fast MRI reconstruction techniques?
(b) What are the recent advancements in the development of loss functions and training with adversarial networks for MRI reconstruction?
(c) What are the recent advancements and potential implications of MRI reconstruction applications, including non-Cartesian reconstruction, super-resolution, joint learning for coil-sensitivity and sampling, quantitative mapping, and MR fingerprinting?
(d) What are the key research directions and unresolved challenges that need to be addressed to further advance the field of fast MRI using DL-based reconstruction networks, including issues related to robustness, accuracy, generalizability, data acquisition, model interpretability, and deployment in clinical settings?

### 1.5. Contributions

This SLR on DL-based MRI reconstruction models offers the following significant contributions:

(a) DL Reconstruction Architectures: This SLR comprehensively explores various architectures utilized in deep MRI reconstruction, including residual learning, image representation encoders and decoders, data-consistency layers, unrolled networks, learned activations, attention modules, plug-and-play priors, diffusion models, and Bayesian methods.
(b) Loss Functions and Training with Adversarial Networks: This SLR emphasizes the use of loss functions and training with adversarial networks in enhancing deep MRI reconstruction methods. It discusses how novel loss functions tailored to specific imaging objectives and training with adversarial networks techniques have led to improved reconstruction performance and enhanced preservation of clinically relevant features.
(c) Exploration of MRI Reconstruction Applications: Various MRI reconstruction applications are also explored, including non-Cartesian reconstruction, super-resolution, joint learning for coil-sensitivity and sampling, quantitative mapping, and MR fingerprinting. These applications demonstrate the versatility and potential of DL models in addressing different challenges in MRI.
(d) Future Insights: The paper offers valuable insights into future directions for fast MRI research, highlighting potential areas for further advancement, including robust generalization and artifacts handling. Researchers and developers can benefit from SLR’s guidance to enhance MRI quality and accelerate acquisition speed in their ongoing efforts.

The remaining summary for the paper is as follows: Section 2 examines papers improving deep MRI reconstruction methods. Section 3 discusses papers improving reconstruction-related MRI applications. Section 4 presents publication trends, challenges and future outlook, and responses to research questions. Finally, Section 5 concludes the paper by summarizing the findings and emphasizing the significance of discussed approaches in MRI reconstruction.

## 2. Papers Improving Deep MRI Reconstruction Methods

DL methods play a vital role in MRI reconstruction of undersampled k-space data by learning advanced prior information that estimates the missing k-space information. These architectures enable the development of fast and accurate reconstruction techniques that surpass the limitations of traditional methods. By automatically learning hierarchical representations and capturing complex relationships, DL models effectively reconstruct high-quality MR images from undersampled k-space data. Their ability to generalize to diverse datasets and adapt to different imaging conditions enhances their applicability in clinical settings. Overall, DL architectures have revolutionized MRI reconstruction, leading to improved reconstruction speed, MRI quality, and robustness, and holding great promise for advancing medical diagnosis and treatment.

The remaining section explores various aspects of DL methods for MRI reconstruction, including details of the network construction, ranging from kinds of convolutional layers to attention models, and training configuration, ranging from loss functions to data preparation. These components are key to successful DL as MRI image reconstruction methods.

### 2.1. MRI-Specific Aspects of DL Methods

When DL methods are used for MRI image reconstruction, there are several problem-specific details that need to be taken into consideration. For example, the voxels of MRI images are usually complex-valued and the acquired MRI data, measured in the k-space, may have more relevant information than the spatial distribution of the proton density itself. MR is a very dynamic and complex system, and while the data is captured, several things are happening and affecting MR signal, such as the sensitivity of the coils, the inhomogeneity of the magnetic field, and even the relaxation of the resonant spins. Because of this, some DL methods for MRI reconstruction were modified to recover the entire k-space, instead of only the final images. More recent approaches exploit both domains, k-space and image domains. Here, we list some DL methods that exploit k-space, dividing them into two types: k-space and dual.

The k-space domain uses the original acquired domain, trying to learn models that preserve the complex-valued data organized in the frequency domain. Models operating here directly learn mappings between undersampled and fully-sampled k-space, exploiting structures and features used to represent k-space data [9]. Dual approaches combine image and k-space domains, leveraging spatial context and frequency information [46]. The choice of domain depends on data, resources, and goals. Each domain offers unique advantages for DL-based MRI reconstruction.

Table 2 shows a comparative analysis of input domain-based MRI reconstruction models. Different input domain-based models are discussed such as self-calibrating nonlinear reconstruction models, deep generative models, dual-domain recurrent networks, and DL inverse problem solvers. While these models achieved significant performance, but have certain limitations, such as limited evaluation of clinical data and reduced interpretability.

### 2.2. DL Reconstruction Architectures

MRI reconstruction poses challenges that can be addressed by incorporating diverse architectural components and approaches, resulting in enhanced image quality, robustness, and reconstruction efficiency. These architectures include residual learning, encoder/decoder priors, data-consistency layers, unrolled network structure, and attention modules, among other contributions. Researchers frequently combine these methods and innovate new variations to attain superior outcomes in medical imaging applications.

#### 2.2.1. Residual Learning

In MRI reconstruction, when network depth increases, DL models face challenges such as vanishing or exploding gradients, which lead to poor performance. To overcome this problem, researchers have developed residual learning (also called skip connections). By learning residual mappings instead of complete transformations, skip connections mitigate the problem of vanishing gradients and improve training convergence. Thus, in MRI reconstruction, the network can learn to distinguish undersampled MRIs from their ground truth images. Skip connections allow information from early layers to bypass multiple transformations and propagate directly to later layers. Through this mechanism, high-quality MRIs can be reconstructed by adding residual details to undersampled MRIs [53,54,55,56,57,58,59,60]. In MRI reconstruction, residual learning offers a number of advantages such as:

(a) Alleviating the vanishing gradient problem, ensuring faster convergence and higher performance for deep networks.
(b) Improving feature propagation and model’s ability to reconstruct fine details.
(c) Learning the discrepancy between the inputs and outputs, simplifying the task for the networks.
(d) Enhancing the expressiveness and modeling capability of large networks with multiple layers.

**Figure 3:**
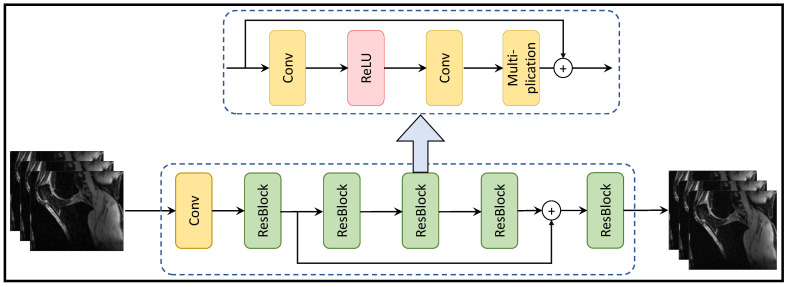
Residual Learning-based MRI Reconstruction Process (adapted with changes from [61]).

Figure 3 depicts the residual learning-based MRI reconstruction process, comprising residual blocks with a sequence of a convolution layer, ReLU activation, another convolution layer, and a multiplication operation. This architecture leads to enhanced MRI reconstruction quality, faster convergence, and efficient memory utilization. Additionally, the model exhibits robustness to noise, adaptability to diverse data distributions, and scalability for varying image sizes.

Table 3 presents a comparison of residual learning-based deep MRI reconstruction techniques, highlighting their contributions and limitations. These models encounter challenges including limited generalization to diverse imaging settings, difficulties in handling artifacts, and high computational resource requirements. Despite these challenges, these techniques demonstrate potential for enhancing MRI reconstruction and advancing the field of medical imaging.

#### 2.2.2. Image Representation Using Encoders and Decoders

**Figure 4:**
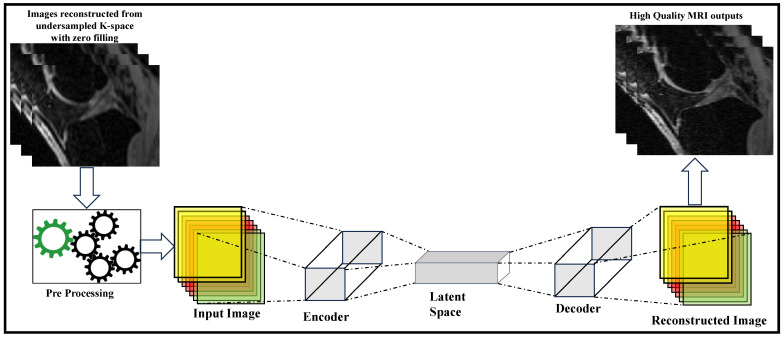
Encoder-Decoder-based MRI Reconstruction Method (adapted with changes from [62]).

Encoders and decoders are standard methods to transform signals and images into arbitrary forms where their structures can be easily represented or learned. The encoder is usually used to convert the signals or images to a different representation, where the features are easily seen and manipulated, while the decoder converts it back to its original format (refer Figure 4). Image reconstruction algorithms have exploited this structure with human-designed filters as encoders and decoders to obtain more effective feature representations for years. But DL methods have been more effective in learning these feature representations from sampled data.

Sun et al. [63] reconstructed the multi-contrast CS-MRI using Deep Information Sharing Network (DISN). Data fidelity units and feature-sharing units were cascaded and densely connected within DISN. There were the same feature maps for all multi-contrast MRIs in the feature-sharing units. In order to facilitate information sharing at different levels, dense connections were used. Zeng et al. [64] reconstructed CS-MRI via Very Deep Densely Connected Network (VDDCN). The network consisted of blocks that are densely connected to each prior block. The blocks were composed of recursive feature extraction modules, fusion sub-blocks, and data-consistency layers. Liu et al. [65] provided an Iterative Feature Refinement Network (IFR-Net) for CS-MRI. In this model, the feature refinement operator and regularization parameter were trainable. It also generalized the sparsity-enforcing operator by utilizing CNN-based inversion blocks.

Sun et al. [66] implemented Deep Error Correction Network (DECN) for CS-MRI. It used three modules such as guide, error correction, and data fidelity to overcome CS-MRI inversion problems. Qiu et al. [67] proposed a deep neural network inspired by the iterative shrinkage-thresholding algorithm with data consistency (NISTAD) for fast undersampled MRI reconstruction. Guo et al. [68] designed an Over-and-Under Complete Convolutional Recurrent Neural Network (OUCR) for MRI reconstruction. Undercomplete branches were used to emphasize low-level features while preserving global structures in OUCR. Feng et al. [69] implemented a Dual-OctConv for fast parallel MR reconstruction. It learned multi-scale spatial-frequency features from real and imaginary components, reducing spatial redundancy. Dual-OctConv utilized octave convolutions to capture richer representations and performed inter-group information exchange for contextual aggregation.

Shangguan et al. [70] proposed a Deep fusion connection network (DFCN) to enhance CS-MRI reconstruction quality. DFCN effectively utilized correlation information between adjacent slices through dense connections and squeeze-and-excitation blocks. Long skip connections were used to avoid gradient explosions and limit low-frequency information flow. Tong et al. [71] designed a Hybrid Image-Wavelet Domain Reconstruction Network (HIWDNet) for fast MRI reconstruction, operating in both transform and image domains. Additionally, the region adaptive artifact removal module was incorporated to effectively eliminate aliasing artifacts in large areas. Wang et al. [72] proposed the Detail and Structure Mutually Enhancing Network (DSMENet), which enhanced structure and detail information through UNet, detail feature refinement module, and bidirectional alternate connections.

Jin et al. [73] proposed a method for reducing ghost artifacts in undersampled MRI scans. They utilized a complex difference transform and a Sparse Complex-valued U-type CNN (SCU-Net) trained on sparse complex-valued edge maps to perform deghosting. The final complex MRIs were obtained by applying k-space inverse filtering to the predicted deghosted edge maps. Zhou et al. [61] developed a deep Residual Non-Local Fourier Network (RNLFNet), which incorporated non-local Fourier attention and residual blocks. The model effectively learned information from both the spatial and frequency domains, capturing local details and global context between degraded MR images and ground truth image pairs, leading to improved reconstruction quality. Dai et al. [74] utilized a Gradient-enhanced Fusion Network (GFN) to reconstruct CS-MRI. The network employed dilated convolution and dense residual learning to extract features, while gradient maps provided structural information. The gradient priors were used to preserve contrast and edge information.

Table 4 provides a comparison of encoder and decoder-based models for MRI reconstruction. The models offer improved reconstruction quality, robustness, and reduced errors. However, challenges such as limited generalization to unseen data, interpretability issues, and computational efficiency need further consideration.

#### 2.2.3. Data-Consistency Layers and Unrolled Networks

Data-consistency layers and unrolled networks are two important components in DL-based MRI reconstruction methods. These components are inherited by classical iterative reconstruction methods. Data-consistency layers enforce consistency between the undersampled k-space data and intermediate versions of the reconstructed image inside the network structure and during the training process. These layers help the network to produce accurate reconstructions, consistent with the measured k-space data. Meanwhile, unrolled networks operate in an iterative manner, where the reconstruction process is unrolled into multiple steps.

**Figure 5:**
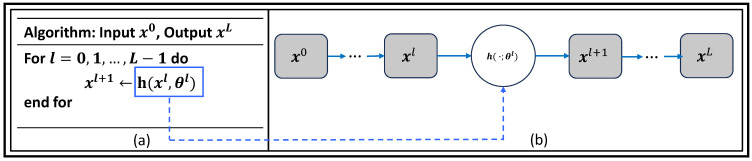
Unrolled networks: Mapping an Iterative Algorithm into a (**a**) Deep Network with (**b**) Trainable Parameters (in Blue) (adapted with changes from [75]).

Figure 5 shows the general idea of unrolling algorithm involves transforming an abstract iterative algorithm into a deep neural network. In this process, each iteration, represented as the function *h* parametrized by $\theta^{l}$, where l=0,1,…,L−1, is mapped into a single network layer. By stacking a finite number of these layers together, we create a deep network. When we feed data through this *L*-layer network, it is equivalent to executing the original iterative algorithm *L* times, but with finite truncation. The parameters represented by $\theta^{l}$, where l=0,1,…,L−1, are learned from real data sets by training the network end to end to optimize its performance. These parameters can either be shared across different layers or vary from layer to layer, depending on the specific demands of an application. In the unrolled deep network (b), the trainable parameters are colored in blue, indicating that these parameters will be adjusted during the training process. The resulting deep network, unrolled from the original iterative algorithm (a), can then be used to perform various tasks, often with improved performance compared to the abstract iterative algorithm alone.

Hammernik et al. [33] utilized Variational Network (VN) to learn a complete reconstruction process for complex-valued multi-channel MR data, eliminating the need for manual parameter tuning. Chen et al. [34] also utilized VN to reconstruct the Single-Shot Fast Spin-Echo MR images. Aggarwal et al. [76] proposed MoDL, an image reconstruction method using CNN-based regularization. It included numerical optimization blocks for complex models and image priors. The variational model-based formulation with shared weights achieved better results in data-constrained settings. Kocanaogullari et al. [77] introduced a Projection based Cascaded CNN (PC-CNN) to reconstruct MRIs. It utilized a projection-based updated data consistency layer with a secondary output to store residual images representing innovation at each stage. Polak et al. [78] developed a joint multi-contrast VN (jVN) approach that leverages shared anatomical structures to improve efficiency and MRI quality.

Wang et al. [79] employed a deep residual complex CNN, called DeepcomplexMRI, for MRI reconstruction. It incorporated the correlation between real and imaginary parts of MRIs and enforced k-space data consistency within its layers. Hosseini et al. [80] used Dense-RNN architecture, derived from the history-cognizant unrolling, for multi-coil MRI reconstruction. They calculated gradient descent steps based on a trainable combination of previous regularization unit outputs. Zhang et al. [81] proposed Total Variation-Inspired Network (TVINet), which incorporated the deep priors with the iterative algorithm. TVINet utilized the primal-dual hybrid gradient algorithm to provide interpretability. Vishnevskiy et al. [82] proposed an approach based on deep VNs, known as FlowVN, for rapid 4D flow reconstruction. The network accurately reconstructed pathological flow in a stenotic aorta in 21 s, allowing for learnable spatiotemporal filter kernels, activation functions, and regularization weights in each iteration.

Aghabiglou and Eksioglu [83] introduced a noise parameter in CNN and UNet architectures, resulting in improved performance of the unfolding structures without a significant increase in complexity. The adaptively calculated noise level parameter at the network’s input leads to enhanced reconstruction performance. Zhang et al. [84] introduced Deartifacting Module (DEMO) to effectively eliminate artifacts in CS-MRI. A robust loss function was derived by augmenting the measurements in the original loss function. DEMO can be flexibly incorporated into both model-based and unrolled deep neural network CS-MRI methods since it is independent of any backbone algorithm. Ottesen et al. [85] implemented the Densely Interconnected Residual Cascading Network (DIRCN) for MRI reconstruction, drawing inspiration from the end-to-end variational network. The method utilized input-level connections and long-range skip connections to enhance MRI quality at high acceleration rates.

Table 5 provides a comparative summary of various data-consistency layers and unrolled networks-based MRI reconstruction methods. These methods have contributed to accelerated MRI reconstruction by incorporating regularization techniques and introducing efficient DL approaches. However, limitations include challenges in generalization to complex patterns and structures in the images, large training data requirements, and computational demands.

#### 2.2.4. Learned Activations and Attention Modules

Non-linear activations play a crucial role in deep networks by selectively focusing on the most relevant features or regions within the input images. It assigns non-linearly weights to pixels, regions, or features, enabling the network to recover elements and features of higher importance. Recently, researchers realized that these non-linear elements can be more efficient if activations are learned. This was seen in [33] where the activation is also learned, instead of fixed, such as in ReLUs. Also, because activations preceded by CNNs only sense locally, researchers investigated architectural structures able to sense features non-locally, giving rise to attention modules. With attention modules, the network is able to improve the accuracy and quality of the reconstructed MRIs, capturing intricate details and subtle structures [86,87]. Although attention modules can introduce computational demands, memory-efficient self-attention modules have been developed to address this limitation, making the integration of attention mechanisms more efficient in MRI reconstruction [88,89]. Overall, the attention module efficiently guides the network’s attention to relevant image content, contributing to MRI reconstruction [90,91,92,93,94,95,96].

Table 6 summarizes various AM-based MRI reconstruction models and their key contributions and limitations. These models leverage attention mechanisms to improve MRI quality and reconstruction performance. For example, the integration of self-attention modules in convolutional layers helps capture long-range dependencies in MRI images. However, a common limitation is the limited generalizability and robustness of the models to different imaging scenarios and acquisition techniques. Another challenge is the reliance on specific assumptions, such as the same coil number or the conversion of multi-channel images into single-channel format. Computational complexity, limited training data, and the need for real data instead of synthesized training data are also highlighted as limitations. Despite these limitations, the models demonstrate promising advancements in MRI reconstruction and provide a foundation for further research and improvements in this field.

#### 2.2.5. Plug-and-Play Priors, Diffusion Models, and Bayesian Methods

In these three approaches, DL networks are learned independently of the reconstruction process. Once trained, the networks are used in a reconstruction algorithm to recover MR image. The main advantage is that the network can be trained with more general data, which is not exactly the same kind of reconstruction. Also, MRI acquisition model is not used for training, so the trained DL prior is general enough to be used in reconstructions with different MRI configurations. The difference among these three approaches lies in the specifics of how the network should be trained and how the reconstruction algorithm is constructed.

Plug-and-play priors are the most general and flexible of these three approaches. The network is trained to replace the image prior of iterative reconstruction algorithms, and it is plugged on the iterative reconstruction method. Yazdanpanah et al. [97] introduced a deep plug-and-play prior framework for parallel MRI reconstruction. An encoder-decoder UNet convolutional network was employed with skip connections as Deep Neural Network (DNN) architecture. This framework not only accelerates MRI acquisition but also significantly enhances the overall image quality. Liu et al. [98] introduced the Regularization by Artifact-REmoval (RARE) framework for MRI reconstruction, which utilizes artifact removal-trained network priors. RARE is applicable in scenarios where fully-sampled ground truth data is unavailable for training. Yang et al. [99] combined low-rank prior and deep-prior to reconstruct CS-MRI. Fast flexible denoising CNN (FFDNet) provided a deep prior, whereas a low-rank prior was obtained using weighted shadow p-norm. In this model, the noise level and weights were automatically determined so that they did not need to be manually set. Hou et al. [100] introduced TRPA, a truncated residual-based Plug-and-play ADMM algorithm for MRI reconstruction using a denoising neural network with CCIN layer. TRPA ensured strict convergence to a fixed point and achieved comparable results. Xie and Liu [101] used Deep Gaussian Denoisers (DGD) to improve CS-MRI reconstruction. DGD network was trained initially on images and subsequently integrated into a plug-and-play framework utilizing a classical momentum strategy and a modified proximal gradient algorithm. Additionally, efficient artifact removal was achieved through the use of a non-local denoiser. Hou and Li [102] designed an iterative IDPCNN model, which combined half-quadratic splitting and CNN for MRI reconstruction. The model offered quick, flexible, and accurate results by incorporating denoising and projection stages.

Diffusion models formulate the reconstruction as a statistical sampling from a learned probability distribution [103,104]. Also closed connected to Bayesian approaches. Essentially, diffusion models consist of progressive steps that modify the prior distribution of the data into a Gaussian distribution. A score network is trained and used as an inverse diffusion, a denoising process with denoising levels controlled by the diffusion steps. The network is trained to extract certain amount of noise, instead of producing a clean image, and later it is used in the iterative algorithm that represents the statistical sampling process.

Gungor et al. [105] accelerated MRI reconstruction using a rapid diffusion prior with an adversarial mapper for efficient image generation. The current drawback of diffusion models is their reconstruction time, which can be several orders slower than iterative algorithms used for CS. In practice, reconstruction times are in the order of 10 min per image, compared to a few seconds of CS reconstruction and less than a second on fast DL reconstruction approaches, such as a VN. In [106], a generative network was utilized as the image prior in a maximum a posteriori (MAP) reconstruction algorithm. In [107], variational autoencoders were proposed to be used as priors in MAP reconstruction. While in [108], denoising autoencoders are used. In [103], score networks are used in reverse diffusion, alternating with data-discrepancy steps, essentially a gradient descent step of data-discrepancy cost. In [104], they propose a similar approach inspired by Bayesian sampling, that is solved with Langevin steps, where reverse diffusion and data-discrepancy are used in the same iteration. They also compute uncertainty maps. In [105,109], they used adversarial networks to train reverse diffusion, together with larger diffusion steps, in order to improve the convergence speed of the approach.

Recently, Bayesian methods have regained prominence in the context of MRI reconstruction, as they inherently provide a framework to manage and quantify uncertainties. For instance, Luo et al. [106] used a deep Bayesian estimation for MRI reconstruction, demonstrating improved performance in managing uncertainty. In a later study, Luo et al. [104] further explored Bayesian MRI reconstruction using diffusion models with joint uncertainty estimation, which further advanced the practical applications of Bayesian methods in this field. Narnhofer et al. [110] also utilized Bayesian uncertainty estimation for variational MRI reconstruction. They used this approach to leverage the power of machine learning algorithms while incorporating the inherent uncertainty present in medical imaging data. Similarly, Khawaled et al. [43] proposed a non-parametric assessment of uncertainty in DL-based MRI reconstruction from undersampled MRI data, demonstrating the benefits of uncertainty assessment in this context. Beyond MRI reconstruction, Bayesian approaches have also been applied in other imaging modalities. Leynes et al. [44] proposed a Bayesian DL method for PET/MRI attenuation coefficient estimation, indicating the flexibility of Bayesian methods in different imaging contexts. Meanwhile, Tanno et al. [45] applied uncertainty modeling in DL for safer neuroimage enhancement, showcasing how Bayesian uncertainty estimation can contribute to safer and more reliable imaging results.

### 2.3. Training

#### 2.3.1. Dataset

Table 7 provides a comprehensive overview of popular MRI reconstruction datasets, encompassing a range of body parts and imaging modalities. The datasets incorporate advanced features such as simulated noise, intensity non-uniformity, pathology, availability of raw k-space data, multi-coil data, different field strengths, multi-center data, and manual segmentations. These features enhance the datasets’ suitability for evaluating and benchmarking MRI reconstruction models. The datasets mentioned, including BrainWeb, FastMRI, IXI Dataset, Calgary-Campinas Public Brain MR Dataset, ACDC Challenge Dataset, and IXI Stroke Dataset, provide ample opportunities for researchers to analyze and refine MRI reconstruction methodologies. Leveraging these datasets can lead to advancements in imaging quality, diagnostic precision, and ultimately enhance the field of MRI reconstruction.

#### 2.3.2. Loss Function

The field of MRI reconstruction is an active area of research, with ongoing developments and exploration of new loss functions. Researchers frequently customize loss functions to match the specific characteristics of the imaging task and desired reconstruction properties. The selection of an appropriate loss function depends on the defined objectives, the MRI quality criteria, and the trade-off between fidelity, perceptual quality, and other desired attributes of the reconstructed images. This process ensures that the chosen loss function aligns with the specific requirements of the MRI reconstruction task, facilitating the production of optimal results. In this section, we analyze several state-of-the-art deep MRI reconstruction techniques, focusing on the choice and design of loss functions, and their impact on the reconstruction performance.

Xuan et al. [117] applied a combination of a cross-modality-synthesis-based registration loss and a reconstruction loss to optimize the spatial alignment network and the multi-modal reconstruction network. Yang et al. [118] improved the reconstruction performance by employing an adversarial loss along with a proposed content loss. The content loss was designed using perceptual loss, frequency domain Mean Square Error (MSE) loss, and pixel-wise image domain MSE loss, resulting in improved reconstruction details. Edupuganti et al. [119] utilized an adversarial loss function aiming to capture and estimate the uncertainty associated with the reconstruction process, thereby offering more reliable and interpretable results.

Jiang et al. [120] integrated perceptual loss, image loss, and frequency loss into the loss function during network training to enhance the preservation of fine structures in the reconstructed images. Li et al. [121] proposed a dual discriminator generative adversarial loss function that integrated holistic image and multi-scale edge information. This aimed to stabilize training, prioritize edge recovery, and enhance reconstruction accuracy, resulting in improved quality and accuracy of the reconstructed images. Zhu et al. [122] proposed DESN, an efficient MRI denoising method based on a neural network approach. DESN utilized a unique network architecture, incorporating encoder and decoder networks with skip connections. The method employed a well-designed loss function, including data fidelity and image quality penalty terms.

Li et al. [88] employed a combination of generative adversarial loss and cyclic data consistency loss, resulting in excellent reconstruction performance even at high under-sampling rates. Quan et al. [35] utilized cyclic loss to enforce data consistency constraints and promote accurate interpolation of undersampled k-space data. Salehi et al. [123] employed Geodesic loss to minimize error and improve the accuracy of 3-D pose estimation in registration applications, enabling more precise alignment and achieving robust performance. Georgescu et al. [124] performed dual loss computations after the upscaling layer and the last convolutional layer, comparing the output to the ground-truth high-resolution image. The inclusion of the intermediate loss encouraged the network to generate more accurate results that closely resembled the ground truth.

Kusakunniran et al. [125] proposed the dual-domain loss, which combined L1 losses in the spatial and frequency domains. This loss function improved reconstruction quality by addressing differences between reconstructed and ground truth MR images, leading to a reduction in aliased artifacts. Wang et al. [126] incorporated an enhanced antagonism loss function to mitigate the generator-discriminator imbalance. It involved adding the discriminator’s discriminant result to the generator loss, along with the ground truth. Zhang et al. [84] employed Huber loss, which balanced robustness and precision by incorporating a hyper-parameter. Huber loss is known for its robustness in handling outliers compared to other loss functions. Tolpadi et al. [127] utilized ROI-specific loss function during network training to enable “ROI-specific optimization” to preserve small clinical features in cartilage and intervertebral discs.

However, the utilized loss functions may face challenges related to generalization and interpretability. To address these concerns, regularization techniques like weight decay and training with adversarial networks can be employed to enhance robustness and generalization. Moreover, incorporating additional metrics such as perceptual loss or structural similarity index can improve interpretability and provide meaningful insights into the reconstruction process.

#### 2.3.3. Training with Adversarial Networks

GANs have revolutionized MRI reconstruction by synthesizing photorealistic images. GANs consist of a generator and discriminator, where the generator, which could be any reconstruction network previously discussed, aims to produce reconstructed MRIs that resemble fully sampled ones, while the discriminator distinguishes between real MRIs and fake ones. Both networks are trained together, but only the generator is deployed for MRI reconstruction. The discriminator is only used during training and acts like a trained evaluator detecting if the images produced by the generator are artificially generated. The training is completed when the discriminator cannot distinguish if the images are real or produced by the generator. GAN-based MRI reconstruction models have shown superior reconstruction performance compared to traditional techniques.

**Figure 6:**
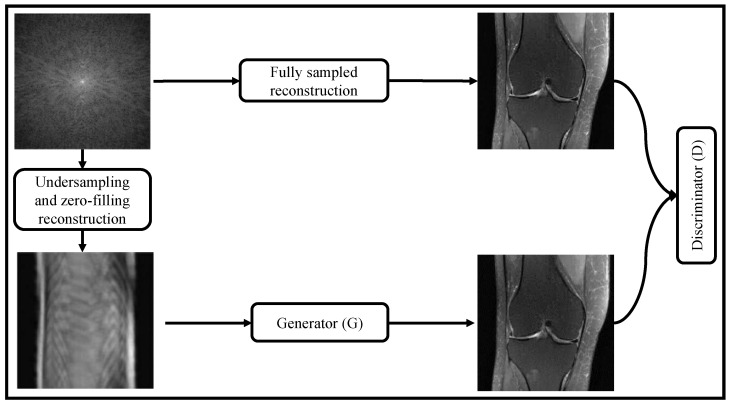
Generative Adversarial Network (GAN)-based MRI Reconstruction Process.

Figure 6 shows GAN-based MRI reconstruction process of reconstructing high-quality MRIs from undersampled data. The method involves several key steps, starting with the data preparation phase, where a dataset of undersampled MRI images and their corresponding fully-sampled ground-truth images is acquired. These undersampled images are obtained through undersampling and zero-filling reconstruction techniques. Thereafter, a GAN architecture is set up for MRI reconstruction, consisting of two main components: a generator and a discriminator. The generator is a deep neural network trained to take the undersampled MRI images as input and generate high-quality, fully-sampled MRI images as its output. It learns the underlying mapping from the undersampled to the fully-sampled images. On the other hand, the discriminator, another deep neural network, acts as a binary classifier. It takes both real (fully-sampled) MRI images and the generated images (output of the generator) as input and aims to distinguish between them, determining whether they are real or fake.

GAN is trained in an adversarial manner, where the generator and the discriminator engage in a two-player minimax game. The generator attempts to produce realistic MRI images that can deceive the discriminator into believing they are fully-sampled, while the discriminator seeks to accurately classify real and generated images. The generator’s loss function is designed to encourage the generated images to be similar to the fully-sampled ground-truth images, while the discriminator’s loss function penalizes incorrect classifications and encourages it to correctly distinguish between real and generated images. GAN is trained iteratively, with both the generator and discriminator updated using backpropagation and gradient descent methods to improve their respective objectives and achieve better reconstruction results.

The remaining section discusses some recently developed GAN-based MRI reconstruction models.

Quan et al. [35] proposed the RefineGAN model for CS-MRI reconstruction, which incorporated GANs, residual networks, and a convolutional autoencoder. By integrating GANs into the framework, the model achieved enhanced reconstruction performance and improved image fidelity. For MRI motion correction, Johnson and Drangova [36] designed the Motion Correction conditional GAN (MoCo-cGAN) network. Dar et al. [37] proposed the reconstructing-synthesizing GAN (rsGAN) for recovering undersampled multi-contrast MRI acquisitions. It incorporated shared high-frequency, low-frequency, and perceptual priors to preserve details and enhance features. Oh et al. [128] used the Optimal Transport CycleGAN (OT-CycleGAN), an unpaired DL method, for accelerated MRI. OT-CycleGAN utilized optimal transport theory and a customized penalized least squares cost to align distributions between different domains. Do et al. [129] proposed X-net and Y-net networks enable effective reconstruction of T1- and T2-weighted MRIs from down-sampled data. The inclusion of a GAN and optimized sampling patterns further enhanced the reconstruction quality.

For better edge restoration and de-aliasing in CS-MRI reconstruction, Li et al. [121] utilized the Edge-Enhanced Dual Discriminator GAN (EDDGAN). EDDGAN stabilized the training process and controlled the hallucination of details by employing a multi-scale edge fusion generator and double discriminator. Zhou et al. [130] utilized a structurally-strengthened GAN with enhanced feature propagation and expression ability by incorporating strengthened connections and residual in residual blocks in its generator. To provide better generalization, Vasudeva et al. [131] proposed a Complex-Valued GAN (Co-VeGAN). In this model, complex-valued weights and operations were explored, and a complex-valued activation function was designed.

Yurt et al. [132] proposed ProvoGAN, a deep generative model for MRI reconstruction that utilized a progressive volumetrization approach. The model sequentially mapped cross-sectional slices optimized for rectilinear orientations, effectively decomposing complex volumetric image recovery tasks. Zhao et al. [133] proposed SwinGAN for MRI reconstruction, which combines GAN and Swin transformer. They utilized a dual-domain generator, considering both the image and frequency domains, with Swin transformer as the backbone to capture long-distance dependencies. Lyu et al. [134] utilized a multi-view transformer-based GAN for the reconstruction of cine MRI. They incorporated cross-view attention to effectively capture spatiotemporal information between adjacent views. To reduce the aliasing artifacts in CS-MRI, Gao et al. [135] implemented the Hierarchical Perception Adversarial Learning Framework (HP-ALF). They reduced the visual perception gap between the overall and regional perspectives by utilizing image information at both the image-level and patch-level. HP-ALF effectively enhanced perceptual quality and minimized MRI distortion.

Table 8 provides a comparative analysis of GAN-based MRI reconstruction models. These models contribute to improving MRI quality and achieving superior performance compared to traditional techniques. However, GANs face challenges such as training instability, slow convergence, limited evaluation of clinical datasets, and lack of interpretability. The effectiveness of GAN-based methods in MRI reconstruction can be further enhanced by incorporating auxiliary penalties and enforcing fidelity in the image or k-space domains.

#### 2.3.4. Other Training Approaches

**Figure 7:**
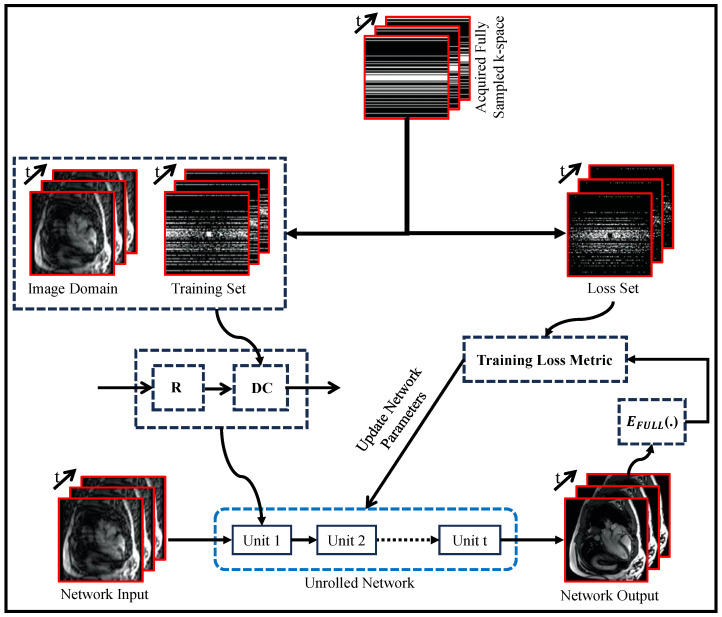
Self-Supervised Training Paradigm for Unrolled MRI Reconstruction Network: Regularizer (R) and Data Consistency (DC) Components (adapted with changes from [137]).

Figure 7 illustrates a self-supervised approach to enhance DL-based MRI reconstruction methods. This approach involves separating the undersampled data into two sets: one for training and the other for validation and loss calculation. By carefully dividing the data in this way, the model gains valuable insights during training, leading to improved performance. This data separation strategy prevents overfitting and enables effective generalization to unseen data. The validation set plays a crucial role in evaluating the model’s performance and guiding the training process for fine-tuning the reconstruction. By combining this data separation strategy with data consistency (DC) and regularization (R) components, the model adapts better to diverse datasets, resulting in robust and accurate image reconstructions from undersampled data.

Yaman et al. [136] developed a self-supervised learning approach for training physics-guided DL-MRI reconstruction without depending on fully sampled reference data. SSDU split acquired k-space indices into two sets, allowing end-to-end training and evaluation of the network using only acquired measurements. Acar et al. [137] proposed self-supervised training for deep neural networks in dynamic MRI reconstruction that enabled the use of more complex models even in the absence of ground-truth data, making it valuable for high spatiotemporal-resolution protocols. Hu et al. [138] applied a parallel network training approach using self-supervised learning for MRI reconstruction. During model optimization, they utilized two subsets of undersampled data to train two parallel reconstruction networks, thereby improving frequency information recovery. Reconstruction losses were defined on all scanned data points, and a difference loss enforced consistency between the networks. This allowed proper training with only undersampled data. Elmas et al. [139] proposed FedGIMP, a federated learning framework for MRI reconstruction. It leveraged cross-site learning of a generative MRI prior and adaptation with subject-specific imaging operators.

### 2.4. Other Improvements

Murugesan et al. [140] proposed ReconSynergyNet (RSN) and a deep cascade RSN, where RSN blocks are interleaved with data fidelity units. They also used Gradient of Log Feature (GOLF) fusion to provide additional structural information for T2-weighted images using T1-weighted images with shorter acquisition times. Lastly, the Perceptual Refinement Network (PRN) was applied to enhance image fidelity. Ueda et al. [141] studied the MRI reconstruction performance using DL and CS. In this, Advanced Intelligent Clear IQ Engine (AiCE) (i.e., a DL reconstruction method) and Compressed SPEEDER (C-SPEEDER) were utilized to improve MRI quality while reducing noise. It provided better performance than conventional SPEEDER. Thomaz et al. [142] utilized the combination of U-Nets and L1, L2, and TV optimizations to reconstruct the images from highly undersampled MRI data. Genzel et al. [143] showed that standard end-to-end deep learning algorithms for inverse problems are robust against both statistical noise and adversarial perturbations, without the need for complex defense strategies.

The principle of dictionary learning has also found diverse applications, ranging from signal processing and computer vision to medical imaging, particularly in the field of MRI reconstruction. Several studies have explored the integration of deep dictionary learning for improving MRI reconstruction methods. Singhal and Majumdar [144] introduced structured deep dictionary learning for the reconstruction of multi-echo MRIs. By combining DL and dictionary learning, their approach improved the quality and precision of the reconstructed MRIs, enabling better analysis and interpretation of the acquired data. Rai et al. [145] developed an augmented noise learning framework for medical image denoising, specifically focusing on MRI. Their framework integrated dictionary learning techniques to enhance the denoising process, leading to improved image quality and increased diagnostic value of MRI scans.

## 3. Papers Improving Reconstruction-Related MRI Applications

MRI reconstruction applications have a significant impact on advancing the capabilities of MRI imaging. They facilitate faster and higher-quality scans, while also offering valuable quantitative information for clinical and research purposes. These applications comprise non-Cartesian reconstruction, super-resolution, joint learning of reconstruction and coil-sensitivity, joint learning of sampling and reconstruction, quantitative mapping, and MR Fingerprinting.

### 3.1. Non-Cartesian Reconstruction

These MRI reconstruction methods are necessary when non-Cartesian sampling trajectories are used for data acquisition. The Non-Uniform Fast Fourier Transform (NUFFT) plays a critical role in this process by accurately transforming the irregularly sampled non-Cartesian k-space data onto a Cartesian image representation. This transformation is necessary and replaces the fast Fourier transform (FFT) when k-space data is not in the Cartesian grid [146]. Also, NUFFT and its adjoint are usually ill-conditioned operators, reducing the convergence speed of the reconstruction algorithms compared to their Cartesian counterparts [147]. To address this issue, density compensation (DC) techniques have been introduced. Classical MRI trajectories like radial or spiral tend to oversample the center of the k-space, causing a biased weight distribution visible after the adjoint operation. Density compensation addresses this imbalance by applying factors that equalize the contribution of different sample locations, resulting in a more even role for each sample during the adjoint application. By incorporating density compensation, non-Cartesian MRI reconstruction can mitigate artifacts and enhance the quality and fidelity of the reconstructed images [148,149].

The study conducted by Ramzi et al. [148] emphasized the importance of DC in neural networks for non-Cartesian MRI reconstruction. By incorporating a dynamic DC mechanism, the study addressed the issue of uneven k-space weighting, resulting in improved image quality. Notably, this work introduced the first network in the literature that adapts to different sampling densities, highlighting the significance of DC in achieving satisfactory results. The benchmark in this study involved the utilization of emulated single-coil k-space data for evaluation purposes. In their subsequent work [149], they extended their findings to non-Cartesian multi-coil MRI 2D and single-coil MRI 3D settings. Chen et al. [150] utilized a method called preconditioned gradient descent (PGD-DC) for DC, addressing the uneven weighting of the radial k-space data during the MRI reconstruction process. Dwork et al. [151] presented an algorithm for generating density compensation values from a set of Fourier samples. The algorithm considered the point spread function over an entire rectangular region in the image domain. This algorithm demonstrated broader applications in iterative reconstruction algorithms and neural network system models. Wang et al. [152] introduced the parallel non-cartesian spatial-temporal Dictionary Learning Neural Networks (stDLNN) for accelerating 4D-MRI reconstruction. Their method leveraged the power of DL and dictionary learning to expedite the reconstruction of 4D MRI data, enabling real-time visualization and analysis of dynamic processes.

### 3.2. Super-Resolution

SR techniques are extremely useful for MRI. Scanner operators could set the scans for very high resolution, but in practice, as voxel size is reduced SNR is also reduced, limiting the smaller voxel size possible to be obtained in the scanner. In this sense, producing high-resolution images from relatively low-resolution data can solve this problem. Another advantage is faster acquisition, since time is not spent in the acquisition of these high-frequency and low SNR components. Another important application for SR techniques is improving slice thickness. In many 2D scans, the slice thickness cannot be reduced. In this case, SR can be applied to artificially increase the number or slices with finer thickness [62].

Chaudhari et al. [153] developed DeepResolve, a 3D CNN network that aimed to generate high-resolution thin-slice images while reducing scan time. It outperformed tricubic interpolation, Fourier interpolation, and sparse-coding SR in terms of image quality metrics. Zhao et al. [154] investigated SMORE, a self-supervised SR algorithm specifically developed for MRI. SMORE did not rely on external training data and was tailored for MRI acquisitions with high in-plane resolution but low through-plane resolution. Shi et al. [155] implemented Fixed Skip Connection Wide Network (FSCWN) for SR. It utilized the combination of shallow network-based local residual learning and global residual learning in a progressive wide network to capture and preserve fine details for better reconstruction.

Lyu et al. [156] developed a Deep Ensemble Learning Network (DELNet) for SR, combining multiple SR models and GANs. The approach achieved superior artifact suppression and enhanced MRI details compared to individual GANs. Masutani et al. [157] assessed CNNs for their ability to generate single-frame (k) and multi-frame (kt) SR images. Two shallow networks, k-SRNet and kt-SRNet, were employed, along with two deeper networks, k-UNet, and kt-UNet, for this purpose. Ferdian et al. [158] presented 4DFlowNet, a DL model combined with computational fluid dynamics, enabling SR in 4D flow MRI. Their approach improved flow estimation and enhanced the understanding of fluid dynamics. Sarasaen et al. [159] presented a SR model for dynamic MRIs, using prior knowledge-based fine-tuning. It utilized a 3D UNet with perceptual loss, trained on a benchmark dataset and fine-tuned with subject-specific static high-resolution MRI.

Lin and Zihao [160] presented a magnitude-image based CNN model with a data consistency layer, referred to as DC-CNN, for SR in MRI. It was demonstrated that their method enhanced the quality of MRIs without relying on raw k-space data. Shit et al. [161] developed SRflow, a DL-based SR model for 4D flow MRI. They utilized a CNN to learn the inter-scale relationship of the velocity vector map. This led to enhanced spatiotemporal vector field resolution, enabling more precise quantification of hemodynamics. Iwamoto et al. [162] introduced an unsupervised SR model, DEGRNet, using deep external learning and a guided residual dense network. The guided CNN utilized HR images of a different modality to enhance LR image resolution within the same subject. Rudie et al. [163] conducted a clinical assessment of SR for 3D volumetric brain MRI, utilizing a DL-based model for denoising and resolution enhancement. Their focus was on optimizing scan time while preserving image quality and SR for specific image types. Qiu et al. [164] designed a Progressive Feedback Residual Attention Network (PFRN) to enhance the detailed information and visual quality of cardiac MRI. Feature extraction and retention, progressive feedback modules, and MS-SSIM-L1 loss function contributed to better MRI quality and reconstruction.

Table 9 summarizes various SR models in MRI along with their main features and limitations. The models include DeepResolve, DDCN, SMORE, DELNet, SRNet & UNet, 4DFlowNet, 3D UNet, DC-CNN, SRflow, DEGRNet, 3D CNN, and PFRN. These models offer benefits such as improved resolution, enhanced image quality, and compatibility with diverse medical imaging modalities. However, they also have limitations, such as increased computational complexity, potential overfitting, sensitivity to network architecture and hyperparameters, and the need for further evaluation of diverse clinical datasets. Additionally, limitations related to the specific applications, interpretability of learned features, and limitations in addressing smaller and more subtle lesions were noted in some models.

Despite these limitations, the studied SR models show promising potential for enhancing MRI quality and supporting clinical decision-making. Future research efforts should focus on addressing the identified limitations, refining the models’ performance, and investigating their practical utility in clinical settings.

### 3.3. Joint Learning: Coil-Sensitivity and Reconstruction

Joint learning of coil sensitivity and reconstruction refers to simultaneously optimizing the estimation of coil sensitivity maps and the image reconstruction process in MRI. This approach integrates the two tasks, leveraging the mutual information between them. By jointly learning, it is possible to improve image quality, especially in scenarios with complex coil sensitivity variations or artifacts. Deep learning techniques are often employed to directly estimate the coil sensitivity maps and perform image reconstruction in a single step. Joint learning enhances the efficiency, accuracy, and robustness of MRI reconstruction, leading to improved image quality and diagnostic capabilities.

Sriram et al. [168] designed GrappaNet for multi-coil MRI reconstruction, integrating neural networks and GRAPPA to achieve scan-specific reconstruction. The reconstruction process was performed jointly across all complex-valued views captured during the parallel imaging process, allowing the network to effectively leverage all available information. Sriram et al. [169] introduced end-to-end VNs for multi-coil MRI reconstruction, addressing the challenge of the unknown forward process by estimating sensitivity maps within the network and learning fully end-to-end. Jun et al. [170] implemented Joint-ICNet, a Joint Deep Model-based MR Image and Coil Sensitivity Reconstruction Network. It jointly reconstructed MR images and estimated coil sensitivity maps from undersampled multi-coil k-space data using an unrolled network architecture.

Peng et al. [171] used CNNs to estimate coil sensitivity functions in MRI by leveraging information from previous scans. The trained networks effectively mapped the initial sensitivity to high-resolution counterparts. Additionally, sensitivity alignment techniques were employed to mitigate geometric variation. Yiasemis et al. [51] proposed a multi-coil MRI reconstruction approach using recurrent variational networks. The method jointly trains the coil sensitivity and reconstruction network, refining k-space data (observation domain) to achieve high-quality reconstructions from highly accelerated MRI data. Zhang et al. [172] proposed a method in which they simultaneously learned coil-sensitivity and reconstruction for accelerated multi-coil MRI using a VN with explicit feature fusion.

### 3.4. Joint Learning: Sampling and Reconstruction

Joint learning of sampling and reconstruction in MRI involves simultaneously optimizing the sampling pattern or density and the image reconstruction process. This approach leverages data-driven techniques, such as deep learning, to learn the optimal sampling pattern directly from the data. By jointly learning, the algorithm improves the trade-off between acquisition time and image quality, leading to more efficient and accurate reconstructions. Deep learning frameworks train networks to estimate the optimal sampling pattern and perform reconstruction, capturing complex dependencies. Joint learning enhances image quality, reduces acquisition time, and improves overall MRI efficiency.

Zhang et al. [173] introduced RecNet, a reconstruction network that generated MRI reconstructions and uncertainty predictions. RecNet was trained to optimize for both objectives simultaneously. An evaluator network for active acquisition was also proposed, which could recommend optimal k-space trajectories for MRI scanners and effectively reduce uncertainty. Bahadir et al. [174] implemented LOUPE (Learning-based Optimization of the Under-sampling PattErn), a method that simultaneously tackled the problems of optimal under-sampling and image reconstruction. By training a neural network on full-resolution MRI scans, LOUPE generated data-dependent optimized under-sampling patterns, resulting in superior reconstruction quality even at high acceleration rates. LOUPE framework was further extended by Zhang et al. [175] with binary stochastic k-space sampling for in-vivo data, using a modified unrolled optimization network. Learned optimal sampling pattern outperformed hand-crafted patterns with better reconstruction results. Aggarwal and Jacob [176] introduced a continuous strategy for joint optimization of sampling patterns and CNN parameters using a multichannel forward model with continuously defined sampling locations, which improved image quality in deep learning reconstruction algorithms. Weiss et al. [177] introduced PILOT (Physics-Informed Learned Optimized Trajectories), a deep-learning-based method for joint optimization of hardware-viable k-space trajectories. It integrated acquisition parameters and constraints into the learning pipeline to optimize image reconstruction networks simultaneously. Zibetti et al. [178] proposed an alternating learning approach for accelerated parallel MRI, where the sampling pattern and parameters of VN were simultaneously learned. Wang et al. [146] proposed a joint optimization approach for fast MRI, optimizing reconstruction methods and sampling trajectories together using B-spline kernels and multi-scale optimization. Radhakrishna and Ciuciu [179] proposed PROJeCTOR, a joint learning approach that optimized both k-space trajectories and image reconstruction simultaneously. Using a projected gradient descent algorithm, PROJeCTOR learned k-space trajectories in a data-driven manner while adhering to hardware constraints during training.

### 3.5. Quantitative Mapping

End-to-end mapping of MR parameters has been an active area of research in recent years, with a focus on developing efficient and accurate techniques using DL models. These models aim to directly map acquired MRI data to quantitative parameter maps, bypassing complex and time-consuming processing steps.

**Figure 8:**
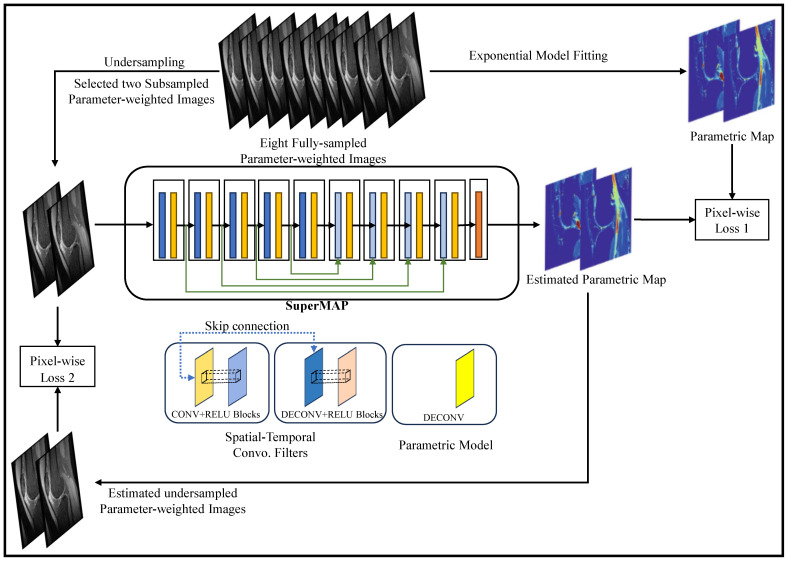
Diagrammatic Flow of SuperMAP-based End-to-End Quantitative Mapping (adapted with changes from [39]).

Figure 8 shows SuperMAP’s training approach which provides a highly effective alternative to derive quantitative maps from undersampled data. By employing eight fully sampled parameter-weighted images, it generates the necessary training data. The SuperMAP network is constructed with multiple skip connections, enabling it to proficiently learn and accommodate variations between input and output data. Each network block is equipped with 64 filters, using a kernel dimension of 3. Throughout the training phase, two loss functions are utilized: Loss1, which optimizes the parametric maps, and Loss2, which ensures data consistency by comparing the generated results with the actual measurement data. This comprehensive training strategy empowers SuperMAP to achieve accurate and reliable quantitative map reconstruction even from limited data samples.

One notable technique is MANTIS (Model-Augmented Neural neTwork with Incoherent k-space Sampling) [38], which combined incoherent k-space sampling with a model-augmented neural network. By leveraging the power of DL, MANTIS achieved high-quality parameter maps from highly undersampled MRI data, enabling rapid and accurate quantification. Relax-MANTIS [180], an unsupervised DL framework, took the concept further by extracting latent maps without relying on reference data. This reference-free approach allowed for efficient parametric mapping and eliminated the need for additional acquisition of reference scans. To enhance the speed of parameter mapping, high-performance rapid MR parameter mapping using model-based deep adversarial learning [41] combined model-based MRI reconstruction and deep adversarial learning. This approach leveraged the strengths of both techniques to achieve fast and accurate parameter mapping.

For simultaneous mapping of multiple parameters, ultra-fast simultaneous T1rho and T2 mapping using DL [181] presented a DL-based approach. By training a neural network on multi-contrast images, this method enabled rapid acquisition and accurate mapping of both T1rho and T2 relaxation times in a single step. In dynamic imaging, kt SANTIS (Subspace augmented neural network with incoherent sampling) [42] utilized subspace learning and incoherent sampling to reconstruct dynamic MR images and efficiently map dynamic parameters. SuperMAP (Deep ultrafast MR relaxometry with joint spatiotemporal undersampling) [39] focused on joint spatiotemporal undersampling to enable rapid acquisition and accurate mapping of relaxation parameters. By combining DL with joint undersampling, SuperMAP achieved ultrafast mapping without compromising quality.

These end-to-end mapping techniques demonstrate the potential of DL in achieving rapid and accurate quantification of MR parameters. By directly mapping acquired data to quantitative maps, these approaches streamline the process, improve efficiency, and hold great promise for advancing clinical diagnosis and treatment planning in the field of MRI.

### 3.6. MR Fingerprinting

Quantitative imaging protocols serve as vital clinical tools, offering objective and precise measurements in the field of medical imaging. Among these protocols, Magnetic Resonance Fingerprinting (MRF) emerges as a powerful technique for quantitative MRI. MRF boasts several compelling advantages, including its flexibility, efficiency, and the ability to simultaneously quantify multiple properties of interest. This unique flexibility empowers clinicians to comprehensively assess anatomical and physiological characteristics within a single acquisition, enabling a more holistic understanding of the imaging data. Furthermore, MRF’s efficiency is achieved through specialized pulse sequences and advanced reconstruction models, resulting in reduced scan times and improved patient comfort and workflow.

Thus, MRF enhances the objectivity and accuracy of diagnosis, treatment response assessment, and disease progression monitoring. By harnessing the capabilities of MRF, medical professionals can extract comprehensive and dependable information from MRI scans, ultimately leading to enhanced patient care and informed clinical decision-making. In recent years, there has been significant interest in leveraging DL models to enhance the accuracy, efficiency, and robustness of MRF quantitative mapping.

**Figure 9:**
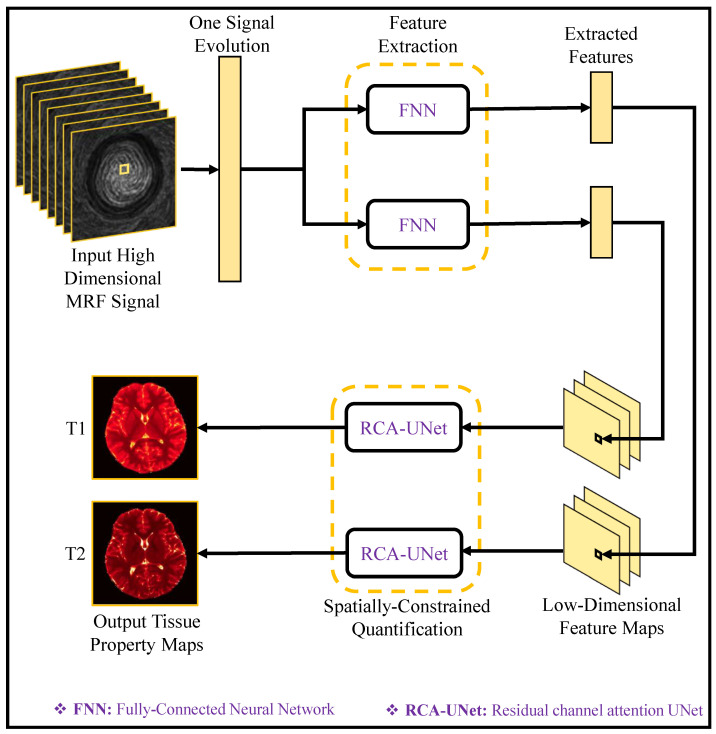
Deep Learning Model for Tissue Quantification in MRF with Spatially Constrained Quantification (SCQ) Methodology (adapted with changes from [182]).

Figure 9 illustrates the deep learning model for tissue quantification in MRF. Initially, the feature extraction module processes each MR signal evolution, extracting a feature vector with reduced dimensions. Subsequently, a spatially constrained quantification module, employing an end-to-end CNN mapping, is employed to estimate tissue maps using the extracted features while preserving spatial information. By employing the spatially constrained quantification (SCQ) method, precise T1 and T2 estimations are achieved, utilizing only a quarter of the originally required MRF signals. This results in a remarkable fourfold acceleration in the brain’s tissue quantification process, highlighting the effectiveness and efficiency of SCQ approach.

Han et al. [40] proposed a fast group matching technique for MR fingerprinting reconstruction. By incorporating a group matching algorithm, they achieved accelerated and accurate mapping of tissue properties in MRF. This approach effectively harnessed the acquired dictionary and enabled efficient quantification. To further improve the speed and reliability of MRF quantitative mapping, DL has been extensively employed. Zhang et al. [183] explored the use of DL for fast and spatially-constrained tissue quantification in highly-accelerated MRF data. Their approach leveraged DL models to accelerate data processing and achieve spatially-constrained quantification, enabling rapid and accurate mapping of tissue properties.

Li et al. [184] focused specifically on the rapid reconstruction of quantitative relaxation maps in MRF using DL models. Their proposed DL-based approach significantly reduced the reconstruction time while maintaining accurate quantification. By accelerating the mapping process, they demonstrated the potential for real-time applications and improved clinical workflow.

In addition to acceleration, DL has been employed to enhance the overall accuracy and robustness of MRF quantitative mapping. Zhao et al. [185] developed a robust sliding-window reconstruction technique that addressed challenges associated with accelerated acquisition in MRF. Their approach effectively improved the reliability and speed of quantitative mapping, enabling more precise characterization of tissue properties. Chen et al. [186] focused on specific aspects of MRF, such as magnetization transfer contrast and chemical exchange saturation transfer imaging. Their DL approach enabled accurate and efficient quantification of these parameters in MRF, contributing to a comprehensive analysis of tissue properties. Golbabaee et al. [187] proposed a method for CS-MRI quantification using convex spatiotemporal priors and deep encoder-decoder networks. By combining dictionary learning with DL models, they achieved accurate quantification of MRI data acquired under compressive settings, enabling efficient storage and analysis of the reconstructed MRIs. As the field of MRF quantitative mapping progresses, DL models continue to evolve. Recent studies have explored the use of complex-valued neural networks [188] to further enhance the accuracy and speed of quantitative mapping in MRF. Zhang et al. [189] introduced a theoretically grounded loss function for network training using Cramer-Rao bound to ensure close to optimal performance in multi-parametric quantitative mapping from complex-valued MRF data, which was undersampled and reconstructed in the low-rank sub-space.

In conclusion, the integration of DL models in MRF quantitative mapping has demonstrated promising results in terms of accelerating the mapping process, improving accuracy, and enabling real-time applications. These advancements in MRF quantitative mapping contribute to enhanced diagnostic capabilities, treatment planning, and monitoring of various diseases and conditions.

### 3.7. Dynamic MRI

Kustner et al. [190] developed 4D CINENet, a deep learning-based reconstruction network for prospectively undersampled 3D Cartesian CINE imaging. The network utilized an unrolled optimization algorithm with complex-valued convolutions and intermittent data consistency blocks to handle the input data effectively. Kofler et al. [191] proposed a deep supervised dictionary learning approach for fast 2D dynamic MR reconstruction. Their method demonstrated the potential of combining DL and dictionary learning to achieve rapid and high-quality reconstruction of dynamic MRI data, facilitating time-resolved analysis of physiological processes. Yoo et al. [192] utilized an unsupervised deep-learning algorithm based on the generalized deep-image-prior approach to optimize the reconstruction network’s weights. Notably, their method achieved successful dynamic MRI reconstruction without relying on prior training or additional data. Huang et al. [193] proposed a dynamic MRI reconstruction approach that infused motion information using deep neural networks. They decomposed the motion-guided optimization problem into a dynamic reconstruction network, motion estimation, and motion compensation components, resulting in improved reconstruction quality. Schlemper et al. [194] presented a deep cascade CNN approach for 2D MR image reconstruction using Cartesian sampling. The network demonstrated strong generalization capabilities and could be trained with various undersampling masks. It achieved high-quality reconstructions in real-time, with each image reconstructed in just 23 ms.

## 4. Discussion

In the discussion section, we examine the importance of datasets in DL-based MRI reconstruction and emphasize the need for diverse and well-curated datasets to enhance model performance and generalization. We also discuss the challenges faced in this domain, such as limited data availability, interpretability of DL models, and integration into clinical practice.

### 4.1. Evaluating DL Reconstruction

The most commonly used performance metrics are Signal-to-Noise Ratio (SNR) [195,196], Contrast-to-Noise Ratio (CNR) [197], Structural Similarity Index (SSIM) [198], Peak Signal-to-Noise Ratio (PSNR) [199,200], Root Mean Square Error (RMSE) [201,202,203], Normalized Root Mean Square Error (NRMSE) [200], and Edge Preservation Index (EPI) [204]. These metrics are commonly used to evaluate the quality, accuracy, and fidelity of MRI reconstruction models. In Table 10, each row corresponds to a specific metric. The “Definitions” column provides a concise description of each metric. The “Formula” column showcases the mathematical formula used to compute the corresponding metric. The “Range” column specifies the valid range of values for each metric, such as a specific interval. The “Desirability” column indicates whether higher or lower values are preferable for each metric.

Metrics like SNR and CNR evaluate MRI quality, while SSIM assesses similarity to a reference image. PSNR quantifies reconstruction quality, RMSE quantifies accuracy, and NRMSE normalizes the RMSE. EPI evaluates edge preservation degree. Understanding these metrics helps researchers and practitioners assess and compare different MRI reconstruction models based on their quantitative performance.

Here, $a_{s}$ shows the average intensity of the signal in the reconstructed MRI ($R_{I}$). $\sigma_{n}$ represents the standard deviation of noise in $R_{I}$. Signal1 ($S_{1}$) and Signal2 ($S_{1}$) are the intensities of the first and second signal or region of interest in $R_{I}$, respectively. $\mu_{x}$ and $\mu_{y}$ represent the average intensity of $R_{I}$ and ground truth image ($G_{I}$). $\sigma_{xy}$ denotes covariance between $R_{I}$ and $G_{I}$. $c_{1}$ and $c_{2}$ are the constants for stability. $\sigma_{x}$ and $\sigma_{y}$ are variances of $R_{I}$ and $G_{I}$. ${\mathrm{MAX}}_{i}$ shows maximum possible pixel value. *N* represents the total number of pixels. $Max_{v}$ and $Min_{v}$ represent the maximum and minimum pixel values in the image. $T_{eb}$ is the total edge blur in $R_{I}$ and $M_{eb}$ is maximum edge blur.

### 4.2. Publication Trends

**Figure 10:**
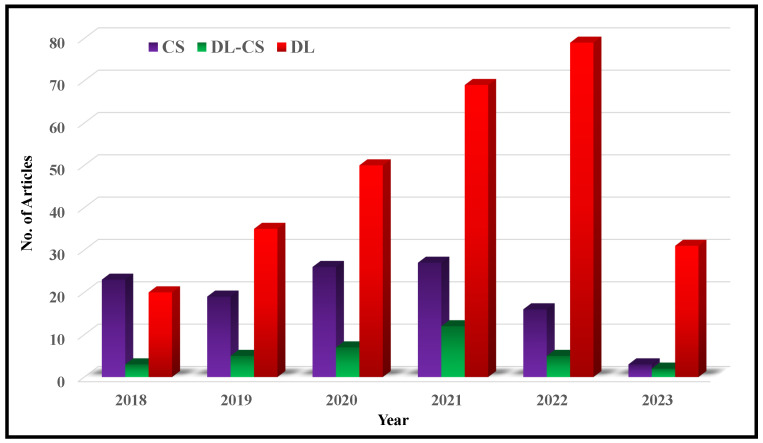
Publication Analysis of DL-based MRI Reconstruction including Compressive Sensing (CS) only, CS combined with Deep Learning (DL), and DL-only (2018–2023).

Figure 10 provides an insightful overview of the publication analysis conducted for an SLR that specifically focuses on the utilization of DL models in combination with CS for MRI reconstruction. The data covers the period from Jan. 2018 to June 2023 and presents the annual publication trends in three distinct categories: CS-only, CS combined with DL, and DL-only.

The results of the analysis reveal a clear indication of the growing interest in DL-based MRI reconstruction. Over the years, there has been an increasing number of papers dedicated to DL-only approaches. The number of CS papers exhibits some variation but remains relatively stable. However, the combination of CS and DL consistently demonstrates an upward trend, showcasing the synergistic potential of these two techniques in MRI reconstruction.

The significant rise in DL-only papers highlights a notable shift in the field toward harnessing the power of DL models for MRI reconstruction. This trend signifies the recognition of DL as a valuable tool in advancing the field of MRI reconstruction.

**Figure 11:**
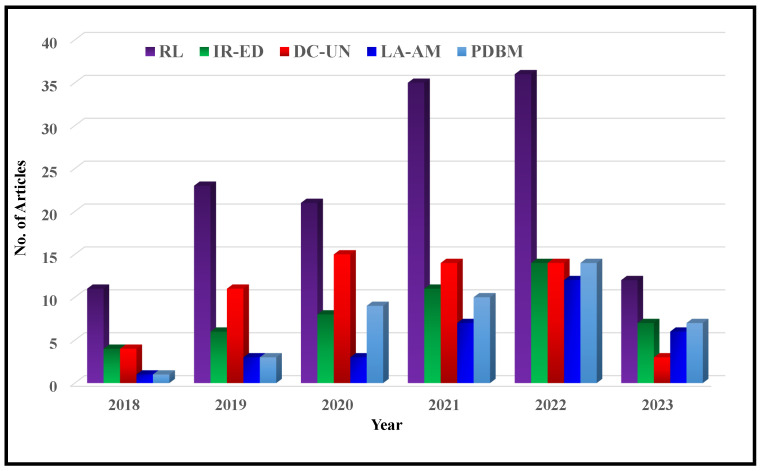
Number of Published Papers on DL Reconstruction Architectures focusing on Residual Learning (RL), Image Representation using Encoders and Decoders (IR-ED), Data-consistency Layers and Unrolled Networks (DC-UN), Learned Activations and Attention Modules (LA-AM), and Plug-and-play Priors, Diffusion Models, and Bayesian Methods (PDBM).

Figure 11 illustrates the analysis of published papers on DL reconstruction architectures, focusing on Residual Learning (RL), Image representation using encoders and decoders (IR-ED), Data-consistency layers and unrolled networks (DC-UN), Learned activations and attention modules (LA-AM), and Plug-and-play priors, diffusion models, and Bayesian methods (PDBM). However, some of these papers utilize more than two approaches at a time. So, for better trend analysis, we have counted them in every category. The number of papers focusing on RL has consistently increased, reaching its peak in 2022 with 36 papers. IR-ED has also shown steady growth, with the highest number of publications in 2022 (14 papers). Similarly, DC-UN and LA-AM have gained attention, reaching their peak in 2022 with 14 papers each. PDBM experienced significant growth from 2018 to 2022, with the highest number of papers (14) in 2022. Overall, these trends indicate the increasing prominence of DL architectures in MRI reconstruction research, with 2022 being the most active year. This suggests a growing interest in the potential of DL architectures to advance MRI reconstruction methodologies.

**Figure 12:**
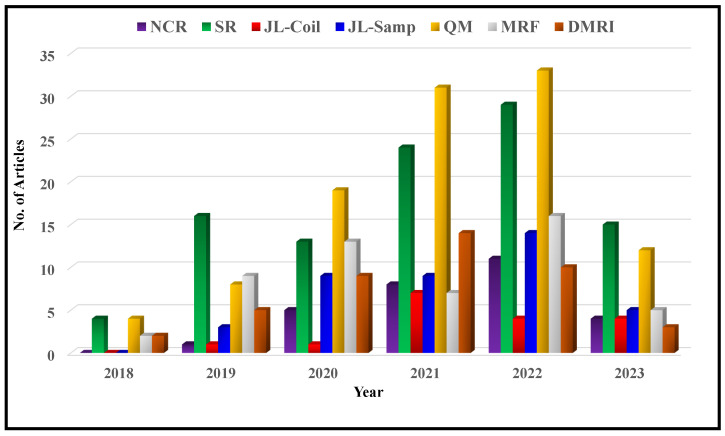
Trends on Improving Reconstruction-Related MRI Applications including Non-Cartesian Reconstruction (NCR), Super-resolution (SR), Joint learning: Coil-sensitivity and Reconstruction (JL-Coil), Joint learning: Sampling and Reconstruction (JL-Samp), Quantitative Mapping (QM), MR Fingerprinting (MRF), and Dynamic MRI (DMRI).

Figure 12 illustrates the publication trends on deep MRI reconstruction applications, including Non-Cartesian Reconstruction (NCR), Super-Resolution (SR), Joint Learning for Coil Sensitivity and Reconstruction (JL-Coil), Joint Learning for Sampling and Reconstruction (JL-Samp), Quantitative Mapping (QM), MR Fingerprinting (MRF), and Dynamic MRI (DMRI). From 2018 to 2023, NCR exhibits a continuous increase in the number of publications, with the highest number in 2022. SR, JL-Coil, and JL-Samp are prominent research areas, consistently garnering attention and experiencing a rise in publications each year. QM and MRF have shown substantial growth, witnessing a notable increase in publications every year. DMRI also maintains steady interest, with a moderate number of publications over the years. Overall, these trends indicate a growing interest and emphasis on advancements in MRI reconstruction applications.

### 4.3. Challenges and Future Outlook

Table 11 highlights the key shortcomings of DL-based MRI reconstruction models and suggests corresponding mitigation strategies. The identified shortcomings include data dependency, limited generalization, black box nature and limited explainability, computational resource requirements, susceptibility to adversarial attacks, challenges in handling artifacts, and the need for effective hyperparameter tuning. To address these limitations, various strategies can be employed, such as data augmentation, transfer learning, domain adaptation, explainable AI techniques, model compression, efficient network architectures, training with adversarial network, data-specific loss functions, and automated hyperparameter tuning techniques. By implementing these mitigation strategies, the performance, robustness, generalization, interpretability, and practicality of DL-based MRI reconstruction models can be improved, thereby advancing their applicability in clinical settings and enhancing their utility in medical imaging.

### 4.4. Responses to Research Questions

The section presents detailed responses to the research questions (refer Section 1.4) that were formulated for this SLR. By addressing the following research questions, this review aims to provide comprehensive insights and understanding into the current landscape of fast MRI reconstruction using DL.

(a) Response to RQ1: Section 2.2 explores the significance of advanced network architectures in fast MRI reconstruction. These architectures, including residual learning, image representation using encoders and decoders, data-consistency layers, unrolled networks, learned activations, attention modules, plug-and-play priors, diffusion models, and Bayesian methods, play a crucial role in improving the efficiency and accuracy of image reconstruction from undersampled k-space data. These advanced architectures contribute to better preservation of small anatomical features in the reconstructed images, making them valuable tools in the development of fast MRI reconstruction techniques.
(b) Response to RQ2: In recent times, significant progress has been made in loss functions and training with adversarial networks for MRI reconstruction, as discussed in Section 2.3. Tailored loss functions designed for specific imaging objectives have improved reconstruction performance and preserved clinically relevant features more effectively. Training with adversarial networks has further contributed to generating realistic and visually pleasing images, mitigating image artifacts and noise in reconstructed MRI scans.
(c) Response to RQ3: In Section 3, we explore recent advancements in MRI reconstruction applications, which have brought about exciting possibilities in the field. Non-Cartesian reconstruction techniques have enabled more flexible sampling patterns, leading to reduced scan times and improved image quality. Super-resolution techniques have achieved higher-resolution imaging, allowing for detailed visualization of anatomical structures. Joint learning for coil-sensitivity and sampling has improved the sensitivity and efficiency of multi-coil MRI data acquisition. Additionally, quantitative mapping and MR fingerprinting techniques have provided valuable insights into tissue properties and physiological processes. These applications hold great potential for advancing clinical diagnostics and research in MRI imaging.
(d) Response to RQ4: Section 4.3 identifies key research directions and unresolved challenges that need to be addressed to advance the field of fast MRI using DL-based reconstruction networks. It highlights the importance of model interpretability to ensure trust and reliability in clinical applications. Furthermore, the review examines the challenges associated with deploying DL models in clinical settings, such as data privacy, regulatory considerations, and integration into existing workflows. By addressing these challenges, future research can drive the field of MRI reconstruction using DL toward practical and impactful clinical applications.

By addressing these research questions, this SLR provides valuable insights into the current state-of-the-art, advancements, and challenges in the field of MRI reconstruction using DL. The findings contribute to a better understanding of existing techniques, their strengths, limitations, and pave the way for future research directions in this rapidly evolving field.

## 5. Conclusions

This SLR comprehensively analyzed the advancements and limitations of DL-based methods in fast MRI, following PRISMA guidelines. The review encompassed both CS and DL methods, providing valuable insights into their respective strengths and weaknesses for revolutionizing fast MRI and improving imaging efficiency.

Throughout the review, various techniques employed in deep MRI reconstruction were highlighted, including residual learning, image representation using encoders and decoders, data-consistency layers, unrolled networks, learned activations, attention modules, plug-and-play priors, diffusion models, and Bayesian methods. Additionally, the use of loss functions and training with adversarial networks was explored to enhance the performance of DL-based MRI reconstruction methods.

The review also explored various applications of deep MRI reconstruction, ranging from non-Cartesian reconstruction to super-resolution, joint learning for coil-sensitivity and sampling, quantitative mapping, and MR fingerprinting. These applications demonstrated the versatility and potential of DL models in addressing different challenges in MRI.

Furthermore, SLR addressed research questions concerning network architectures, input domains, and performance evaluation metrics, offering valuable insights for selecting appropriate techniques and guiding future research directions. It underscored the importance of robust generalization, artifact handling, and the development of explainable AI techniques in the context of fast MRI. Ultimately, the paper contributed to the understanding of DL-based models in fast MRI, serving as a valuable resource for researchers and practitioners seeking to improve MRI quality, accelerate acquisition times, and advance the field of fast MRI. 

## Figures and Tables

**Table 1:** Comparative Analysis of Review and Survey Articles on Deep MRI Reconstruction.

Ref.	Year	Category	CS	SR	QM	MRF
[1]	2020	Review	✓	✗	✓	✓
[21]	2020	Review	✗	✗	✗	✗
[22]	2020	Survey	✓	✗	✗	✗
[23]	2021	Survey	✓	✗	✗	✗
[24]	2021	Survey	✓	✗	✗	✗
[25]	2021	Review	✓	✗	✗	✗
[26]	2021	Review	✓	✗	✗	✗
[27]	2021	SLR	✗	✗	✗	✗
[28]	2021	Review	✓	✗	✗	✗
[29]	2022	Review	✓	✓	✓	✓
[30]	2022	Review	✓	✗	✗	✗
[5]	2022	Review	✓	✗	✗	✗
[2]	2022	SLR	✓	✓	✗	✗
[31]	2023	Review	✓	✓	✗	✗

**Table 2:** Comparative Analysis of Input domain-based MRI Reconstruction Models.

Ref.	Year	Input Domain	Contributions	Unsolved Challenges
[46]	2018	Dual	Implemented KIKI-net, a cross-domain CNN that operates sequentially on k-space, image, k-space, and image to achieve better image reconstruction and minimize aliasing artifacts	Increasing noise levels in data potentially lead to blurred output images and lower PSNR, affecting performance
[9]	2019	k-space	Developed RAKI, a k-space method for non-linear reconstruction of undersampled data from autocalibration signal data, using subject-specific neural networks without extensive training databases	CNN architecture heuristically selected, performance may vary with different network parameters; fixed learning rates in gradient descent algorithm may not be optimal for all applications
[4]	2020	Dual	A dual domain recurrent network was developed to restore both the image and k-space domains, with an embedded T1 prior for enhanced restoration quality	Limited generalization to unseen data and imaging conditions, data scarcity, and the need for large amounts of labeled training data
[47]	2020	Dual	Implemented MRI dual-domain reconstruction network (MD-Recon-Net) to explore the latent relationship between k-space and spatial data	Limited generalization to unseen data and imaging conditions
[48]	2021	k-Space	Employed a residual encoder-decoder network with self-attention layers to achieve adaptive focus and enhance interpolation performance	Potential sensitivity to variations in acquisition parameters and noise levels
[49]	2022	Dual	Utilized complex-valued operations on a cross-domain neural network called the Primal-Dual net (PD-net) for reconstruction and provided an optimal representation of magnitude and phase information in the data	Limited generalization to unseen data and imaging conditions
[50]	2022	Dual	Preserved structure details and removed aliasing artifacts using double-domain GAN	Limited validation on clinical usability, further experiments needed to introduce additional analysis measurements
[51]	2022	k-space	Achieved high-fidelity multi-coil MRI reconstruction using recurrent variational network	Required more memory during training to accumulate gradients for back-propagation during loss function computation
[52]	2022	Dual	Utilized spatial and Fourier domain convolutional layers in an interleaved hybrid domain CNN model, incorporating local residual connections to enhance the reconstruction performance	Still have some residual blur or ringing artifacts that could affect the accuracy of fine details in the reconstructed images

**Table 3:** Comparison of Residual Learning (RL)-based MRI Reconstruction Models.

Ref.	Year	Contributions	Unsolved Challenges
[53]	2018	Used deep residual learning network to learn global artifact patterns, and applied dual frame U-net for artifact correction	Potential blurriness due to L2 loss function
[54]	2019	Implemented multi-scale dilated network using global and local residual learnings to preserve image details	Lack of interpretability and explainability
[55]	2019	Introduced an enhanced recursive residual network by incorporating high-frequency feature guidance, dense connections, and an error-correction unit for superior reconstructions with restored structural features	Network depth balancing, challenges in handling 3D image data, and reliance on precomputed coil sensitivity maps for multi-channel MR data
[56]	2020	Employed sub-band residual learning to enhance high-frequency details in low-resolution MR images and a parallel stream for refined image reconstruction	Does not consider 3D structural and spatial details of MRIs
[57]	2020	Utilized residual learning and attention mechanisms within an encoder-decoder network to transform spherical harmonics coefficients in diffusion MRI	Need more comprehensive evaluation to access the effectiveness and robustness of the network
[58]	2020	Utilized a hierarchical architecture, dense local connections, and global skip-connections to enhance signal synthesis and artifact suppression	Limited data size may limit the generalizability and robustness of the model
[59]	2020	Designed a systematic geometric model using bootstrapping and subnetwork aggregation to increase the expressivity of network	Expressivity improvement scheme was only validated on U-Net and the impact of batch normalization was not analyzed
[60]	2022	Developed denoising of 3D fast spin echo MRIs using spatial-variant noise-relevant residual learning	Network retraining required for changing imaging protocols; potential limitations on real patient data, and longer scan time for ground truth images

**Table 4:** Comparison of encoder and decoder-based models.

Ref.	Year	Network	Contributions	Unsolved Challenges
[63]	2019	DISN	Improved MRI reconstruction quality and robustness to misregistration errors	Limited generalization to unseen data and imaging conditions, lack of interpretability and explainability as black box models
[64]	2019	VDDCN	Made the network easy to train using dense connections and alleviated gradient-vanishing problem	Limited generalization to unseen data and imaging conditions, data scarcity and the need for large amounts of labeled training data
[65]	2019	IFR-Net	Improved network capacity with better feature refinement and fully learned parameters	Limited generalization to unseen data and imaging conditions, prone to overfitting
[66]	2020	DECN	Reduced structural reconstruction errors and improved MRI quality	Lack of interpretability and explainability as black-box models, may lead to artifacts and noise
[67]	2020	NISTAD	Reduced reconstruction time, simplified hyperparameter tuning, and a simpler network architecture with fewer parameters	Not efficient for highly undersampled image sequence reconstruction and might not be realistic enough for real clinical scans
[68]	2021	X-net & Y-net	Reduced number of trainable parameters, leading to a more efficient and streamlined model architecture	Lower computational efficiency due to the incorporation of additional network branches and the increased complexity of the model
[70]	2022	DFCN	Reconstruction quality improved by eliminating aliasing effects utilizing correlation information between adjacent slices	Time-consuming and computationally expensive hyperparameter tuning, may lead to artifacts and noise
[71]	2022	HIWDNet	Achieved accurate cross-domain MRI reconstruction by leveraging image and wavelet domains. Efficiently reconstructed the structure while removing aliasing artifacts.	The complex architecture and intricate interactions of HIWDNet may hinder interpretability
[72]	2023	DSMENet	Enhanced detail and structure information, adapted to diverse MRI scenarios, and offered improved visual effects and generalization. Proved to be a competitive candidate for real-time MRI applications	Complex architecture and intricate interactions of DSMENet limit its interpretability
[73]	2023	SCU-Net	Achieved superior deghosting performance even at high acceleration factors, leading to high-quality complex MRIs	Relied on sparsified complex data and required further investigation into its effectiveness in handling complex anatomical structures and capturing fine details in highly undersampled MRI data
[61]	2023	RNLFNet	Effectively captured long-range spatial dependencies in the frequency domain, leading to enhanced MRI reconstruction	May have limitations when applied to parallel MRI and dynamic MRI
[74]	2023	GFN	Maintain more detailed MR images by capturing edge structures in gradient images	Lack of interpretability and explainability as black-box models, may lead to artifacts and noise

**Table 5:** Data-Consistency Layers and Unrolled Networks-based MRI Reconstruction Models.

Ref.	Year	Network	Contributions	Unsolved Challenges
[33]	2018	VN	Preserved essential features of MR images, including pathologies not present in the training dataset	Suffer from residual artifacts that are particularly evident in the axial sequences
[34]	2018	VN	Provided rapid reconstruction speed of approximately 0.2 s per section	Variation in reconstruction times based on hardware models, the use of constant regularizations, and the absence of fully sampled data
[76]	2018	MoDL	Achieved faster convergence per iteration using numerical optimization blocks for data-consistency and required less training data	Use of many conjugate gradient steps in data-discrepancy layers may lead to increased computational time, possibly reducing reconstruction speed
[77]	2019	PC-CNN	Improved image accuracy by enforcing data consistency and enhanced convergence	Computational complexity, data dependency, limited interpretability, and sensitivity to noise and artifacts
[78]	2020	jVN	Image quality was improved, and blurring was reduced through the learning of efficient regularizers	Generalization to unseen data or different acquisition scenarios
[79]	2020	DeepcomplexMRI	No sensitivity information calculation required for resolving aliasing and channel correlations	High acceleration factors can result in persistent blurriness in the reconstructed MRIs
[80]	2020	Dense-RNN	Showed potential for capturing long-range dependencies among image units	Does not completely address the slow convergence issue inherent in proximal gradient descent methods
[81]	2020	TVINet	Ensured data consistency and preserved the fine details in the reconstructed MRI	Time-consuming and computationally expensive hyperparameter tuning, lack of uncertainty quantification in deterministic predictions
[82]	2020	FlowVN	Achieved accurate reconstructions of pathological flow in a stenotic aorta within a short timeframe of 21 s	Large training data requirement, interpretability
[83]	2022	CNN & UNet	Enhanced unfolding structures without complexity increase, using an adaptively calculated noise parameter for improved reconstruction performance	Suffer from training instability, slow convergence, and limited explainability, which can hinder its practical applicability and interpretability
[84]	2022	DEMO	Efficiently removed CS-MRI artifacts, such as motion, zebra, and herringbone artifacts	High computational requirements, including GPUs, for training and inference
[85]	2023	DIRCN	Used long-range skip connections to improve gradient and information flow	Model trained on retrospective public domain data, needs to be tested on clinically valid prospective data

**Table 6:** Summary of learned activations and attention modules for MRI reconstruction.

Ref.	Year	Contributions	Unsolved Challenges
[86]	2019	Integrating a self-attention module in each convolutional layer to capture long-range spatial dependencies by aggregating features across positions and image regions using weighted calculations	Limited generalizability and robustness of the model to different imaging scenarios and acquisition techniques
[87]	2020	Produced high-quality sum-of-squares images using BarbellNet by incorporating a channel attention mechanism	Same-coil assumption limits practicality, while interpolation or undersampled input leads to information loss and reduced performance
[88]	2021	Enhanced the residual U-net with spatial and channel-wise attention, enabling the network to focus on important information and ignore irrelevant details	Require the conversion of multi-channel MR images into a single-channel format for network input, which increases the workload
[89]	2022	Improved MRI quality and fidelity in reconstruction using a deep adversarial network with cross-attention mechanism to map noise and latent variables onto coil-combined images	Limited performance in capturing the full range of MRI variations and quality due to reliance on unsupervised generative modeling
[90]	2022	Utilized dense and hybrid attention blocks in hybrid attention ResNet to enhance feature extraction and improve MRI reconstruction quality	High computational resource, limited generalization, computationally extensive
[91]	2022	Varying sizes of features were extracted by utilizing a recurrent framework with a non-reduction channel attention block resulting in better reconstruction performance	Struggled to preserve fine structural details in regions with excessive smoothness
[92]	2022	Utilized multi-modality and single-modality reconstruction attention to enable the network to dynamically assign weights and prioritize relevant information from the input modalities	Limited generalization to different settings
[93]	2022	Employed the flow residual attention Unet model, integrating spatial and channel-wise attention blocks, to effectively reduce artifacts and restore velocity information in all encoding directions	Limited number of training samples restrict the exploitation of spatiotemporal or 3D spatial features
[94]	2022	Utilized the fully dense attention CNN to improve generalization by incorporating attention gates in each decoder layer to focus on relevant image features	Dependence on the accuracy of MRI spatial frequencies, which can be a drawback in cases where image features are heavily obscured
[95]	2023	Recovered missing information and preserved realistic structures and textures in MRI reconstructions using a spatial attention selection module and a deep data consistency block	Lack of interpretability, computationally extensive, and limited generalization
[96]	2023	Achieved improved representational ability and captured long-range dependencies by incorporating a squeeze-and-excitation lightweight self-attention module with a dilated depthwise separable convolution dense block	Used synthesized training data instead of real data, which may impact performance on real undersampled MRI data

**Table 7:** Features of Popular MRI Reconstruction Datasets.

Ref	Dataset Name	Body Part	Imaging Modality	Additional Features
[111]	BrainWeb	Brain	T1-weighted, T2-weighted	Simulated noise, intensity non-uniformity, and pathology
[112]	FastMRI	Brain, Knee	T1-weighted, PD-weighted	Large-scale dataset, raw k-space data available
[113]	IXI Dataset	Brain	T1-weighted, T2-weighted, PD-weighted	Multi-center data, various imaging sequences
[114]	Calgary-Campinas Public Brain MR Dataset	Brain	T1-weighted, T2-weighted	Multi-coil data, different field strengths (1.5T and 3T)
[115]	ACDC Challenge Dataset	Heart	Cine-MRI	Multi-center cardiac MRI data with ground truth segmentations
[116]	IXI Breast MRI Dataset	Breast	Dynamic Contrast-Enhanced MRI (DCE-MRI)	Breast MRI data with manual segmentations for studying breast cancer

**Table 8:** Contributions and Limitations of GAN-Based MRI Reconstruction Models.

Ref.	Year	Contributions	Unsolved Challenges
[35]	2018	Under 100 ms, a 256 × 256 MRI can be reconstructed with high quality (over 42 dB in average at 40% sampling rate)	Limited generalization to unseen data and imaging conditions, data scarcity and the need for large amounts of labeled training data
[36]	2019	Reduced motion artifacts and motion blurring consistently by retrospectively correcting MR images with simulated motion	reconstructed MRIs still have a certain amount of smoothness
[37]	2020	Achieved high acceleration factors, successfully recovered pathologies, and could jointly reconstruct and synthesize the target contrast	Large paired datasets are required for training, and further optimization and generalization are necessary to handle diverse multi-contrast imaging scenarios
[128]	2020	Reduced training time and improved network training stability and network generalization	May not fully capture the clinical significance of the phase information
[121]	2021	Balanced edge features against global high-level features for improved reconstruction accuracy	Lack of interpretability and explainability as black box models
[130]	2021	Reconstructed finer MRI texture details and effectively removed artifacts, all while utilizing fewer model parameters	Need to evaluate the generalizability and robustness of the approach across various imaging conditions
[131]	2022	For real-valued activations, a learnable complex-valued activation was developed to solve the transferability issues	Prone to overfitting, lack of interpretability and explainability as black box models
[132]	2022	Captured global context, recovered fine-structural details, and had low model complexity with improved learning behavior	Reliance on fully-supervised training with high-quality datasets, which could be challenging to compile, and the potential challenges in generalizing the model to nonrectilinear orientations
[133]	2023	Efficiently captured both long-distance dependencies and local information	High hardware requirements are associated with the increasing network parameters
[134]	2023	Improved spatiotemporal information was achieved between adjacent views, with a specific focus on reconstructing the local cardiac regions	Not suitable for multi-coil data and requires a large number of network parameters
[135]	2023	Used overall and regional perspectives to remove noise and restore the fine details	Limited generalization to unseen data and imaging conditions, high computational requirements

**Table 9:** Comparative analysis of SR Models.

Ref.	Year	Model	Main Features	Unsolved Challenges
[153]	2018	DeepResolve	Offered the benefit of generating high-resolution thin-slice images while reducing scan time	Focused on magnitude data instead of complex or multichannel data, which may limit the output fidelity
[165]	2018	DDCN	Improved resolution through dense connections, efficient parameter sharing, reduced overfitting	-Increased computational complexity due to dense connections, potential overfitting, sensitivity to network architecture and hyperparameters
[154]	2019	SMORE	Enhanced edges without creating artificial structures and improved both visual and quantitative metrics	Does not address motion artifacts and requires accurate knowledge of the point spread function
[155]	2019	FSCWN	Captured and preserved fine details for better reconstruction using fixed skip connections	Limited generalization to different imaging settings and clinical applicability
[156]	2020	DELNet	Enhanced SR through ensemble learning, leveraging complementary priors	Increased computational complexity due to ensemble size and dependence on diverse ensemble members
[157]	2020	SRNet & UNet	Improved image quality and spatial details in cardiac MRI scans, with the potential for reduced scan time and increased temporal resolution	Lack of a reference standard for accurate comparison, along with limited clinical evaluation and a small patient sample size
[158]	2020	4DFlowNet	Achieved an upsampling factor of 2 and effectively reduced noise in the images	Increased computational complexity and dependence on accurate flow dynamics modeling
[159]	2021	3D UNet	Improved SR of dynamic MRI, fine-tuning for specific applications	Increased computational complexity due to fine-tuning and potential overfitting
[166]	2021	VDR-net	Achieved better resolution of reconstructed MRI images through a Very Deep Residual network (VDR-net) and 2D Stationary Wavelet Transform	Focused on single-image super-resolution and may not have been directly applicable to multi-frame or dynamic imaging scenarios
[160]	2022	DC-CNN	Enhanced the quality of MRIs without relying on raw k-space data	Sensitivity to training data quality and limited interpretability of the learned features
[161]	2022	SRflow	Achieved enhanced spatiotemporal vector field resolution, resulting in more precise quantification of hemodynamics	Generalizability to different datasets and anatomical regions, potential information loss or artifacts during SR, and the complexity of learning vector-field data
[162]	2022	DEGRNet	Utilized clinical image resources without specific HR training images, making it compatible with diverse medical imaging modalities	Limited to 2D super-resolution and potential computational overhead from iterative back projection method
[163]	2022	3D CNN	Clinical assessment of brain SR, improved image quality, accurate structural details	Does not focus on smaller and more subtle lesions especially smaller lesions.
[164]	2023	PFRN	Performed feature extraction directly on LR-MRIs while retaining a significant amount of feature information, enabling the extraction of HF details during the reconstruction process	Assessment on diverse clinical CMRI data is needed to validate PFRN’s generalizability
[167]	2023	CycleGAN	Addressed the limitations of non-blind approaches by utilizing a CycleGAN-based model for domain correction and an upscaling network for reconstruction	Lack of evaluation on clinical datasets

**Table 10:** Performance Metrics for MRI Reconstruction Models.

Metric	Definitions	Formula	Range	Desirability
SNR	Quantifies the ratio of the average signal intensity to the standard deviation of noise in the reconstructed MRI.	$\mathrm{SNR}=20\log_{10}\left(\frac{a_{s}}{\sigma_{n}}\right)$	≥0	Higher
CNR	Measures the difference in intensity between two signals or regions of interest in the reconstructed MRI relative to the standard deviation of noise.	$\mathrm{CNR}=\frac{S_{1}-S_{2}}{\sigma_{n}}$	≥0	Higher
SSIM	Measures the similarity between the reconstructed and reference images in terms of their luminance, contrast, and structural information.	$\mathrm{SSIM}=\frac{\left(2\mu_{x}\mu_{y}+c_{1}\right)\left(2\sigma_{xy}+c_{2}\right)}{\left(\mu_{x}^{2}+\mu_{y}^{2}+c_{1}\right)\left(\sigma_{x}^{2}+\sigma_{y}^{2}+c_{2}\right)}$	[−1,1]	Higher
PSNR	Quantifies the ratio of the maximum possible pixel value to the RMSE, i.e., $\sqrt{\mathrm{MSE}}$ between the reconstructed and reference images.	$\mathrm{PSNR}=20\log_{10}\left(\frac{{\mathrm{MAX}}_{i}}{\sqrt{\mathrm{MSE}}}\right)$	≥0	Higher
RMSE	Calculates the average difference between the pixel intensities in the reconstructed MRI and the corresponding intensities in the original/reference image.	$\mathrm{RMSE}=\sqrt{\frac{1}{N}\sum {\left(R_{I}-G_{I}\right)}^{2}}$	≥0	Lower
NRMSE	Normalizes the RMSE by the range of pixel values in the image.	$\mathrm{NRMSE}=\frac{\mathrm{RMSE}}{Max_{v}-Min_{v}}$	[0,1]	Lower
EPI	Quantifies the preservation of sharp edges in the reconstructed MRI compared to the original/reference image.	$\mathrm{EPI}=1-\frac{T_{eb}}{M_{eb}}$	[0,1]	Higher

**Table 11:** Shortcomings and Mitigation Strategies of DL-based MRI Reconstruction Models.

Shortcoming	Description	Mitigation Strategies
Data Dependency [205,206]	DL models require large labeled training datasets, which may be challenging to obtain, limiting model generalization.	Data augmentation, transfer learning, and domain adaptation techniques can address data scarcity and improve generalization.
Limited Generalization [205,207,208]	Models trained on specific datasets may not perform well on data from different scanners or protocols due to variations in imaging characteristics.	Domain adaptation, model ensemble techniques, and domain-specific regularization methods can enhance generalization across different imaging settings.
Black Box Nature and Limited Explainability [209,210]	DL models lack transparency, interpretability, and the ability to provide detailed explanations for their predictions or reconstruction outputs.	Explainable AI techniques, such as attention mechanisms, interpretability methods, and integration with clinical knowledge or rule-based models, can enhance interpretability and provide explainable outputs.
Computational Resource Requirements [211,212,213]	Training and deploying DL models for MRI reconstruction can be computationally demanding, limiting accessibility in clinical settings.	Model compression techniques, efficient network architectures, and hardware acceleration can help alleviate computational resource requirements.
Susceptibility to Adversarial Attacks [214,215]	DL models can be vulnerable to adversarial attacks, raising concerns about their robustness and reliability.	Adversarial training, input preprocessing (e.g., denoising, smoothing), and defensive mechanisms (e.g., detection, certification) can enhance model robustness against adversarial attacks.
Handling Artifacts and Novel Cases [119,216]	DL models may struggle with complex artifacts that differ significantly from the training data distribution.	Augmenting training data with diverse artifacts, using data-specific loss functions, and incorporating domain knowledge can improve model performance on artifacts.
Hyperparameter Tuning [217,218]	The performance of DL models is sensitive to hyperparameter settings, requiring careful tuning.	Automated hyperparameter tuning techniques (e.g., grid search, Bayesian optimization) and model-specific optimization strategies including metaheuristics can enhance model performance through effective hyperparameter tuning.

## Abbreviations

Following table provides a group of abbreviations along with their corresponding full forms utilized within this SLR. These abbreviations include a variety of ideas, methods, and terms commonly used in the field of fast MRI reconstruction.

ADC	Analog-to-Digital Converter
AE	Autoencoder
AI	Artificial Intelligence
CNN	Convolutional Neural Network
CS	Compressed Sensing
CPU	Central Processing Unit
DL	Deep Learning
FDA	Food and Drug Administration
FFT	Fast Fourier Transform
FID	Fréchet Inception Distance
FOV	Field of View
GAN	Generative Adversarial Network
GPU	Graphics Processing Unit
GUI	Graphical User Interface
HCI	Human-Computer Interaction
k-space	Frequency Domain Data
LSTM	Long Short-Term Memory
MRA	Magnetic Resonance Angiography
MRI	Magnetic Resonance Imaging
NCS	Non-Cartesian Sampling
NMR	Nuclear Magnetic Resonance
PDF	Probability Density Function
PRISMA	Preferred Reporting Items for Systematic Reviews and Meta-Analyses
PSNR	Peak Signal-to-Noise Ratio
QA	Quality Assurance
RAM	Random Access Memory
RF	Radio Frequency
ROI	Region of Interest
RMSE	Root Mean Squared Error
SNR	Signal-to-Noise Ratio
SVM	Support Vector Machine
SSIM	Structural Similarity Index
UNet	U-Net Architecture
